# S-acylation-dependent membrane microdomain localization of the regulatory Kvβ2.1 subunit

**DOI:** 10.1007/s00018-022-04269-3

**Published:** 2022-04-09

**Authors:** Sara R. Roig, Silvia Cassinelli, María Navarro-Pérez, Mireia Pérez-Verdaguer, Irene Estadella, Jesusa Capera, Antonio Felipe

**Affiliations:** 1 Molecular Physiology Laboratory, Departament de Bioquímica i Biomedicina Molecular, Institut de Biomedicina (IBUB), Universitat de Barcelona, Avda. Diagonal 643, 08028 Barcelona, Spain; 2 Imaging Core Facility, Biozentrum University of Basel, 4056 Basel, Switzerland; 3 Department of Cell Biology, School of Medicine, University of Pittsburgh, 3500 Terrace Street, Pittsburgh, PA 15261 USA; 4 Kennedy Institute of Rheumatology, University of Oxford, Oxford, OX3 7FY UK

**Keywords:** Potassium channels, Regulatory subunits, Palmitoylation, Immunological synapse, Lymphocytes

## Abstract

The voltage-dependent potassium (Kv) channel Kvβ family was the first identified group of modulators of Kv channels. Kvβ regulation of the α-subunits, in addition to their aldoketoreductase activity, has been under extensive study. However, scarce information about their specific α-subunit-independent biology is available. The expression of Kvβs is ubiquitous and, similar to Kv channels, is tightly regulated in leukocytes. Although Kvβ subunits exhibit cytosolic distribution, spatial localization, in close contact with plasma membrane Kv channels, is crucial for a proper immune response. Therefore, Kvβ2.1 is located near cell surface Kv1.3 channels within the immunological synapse during lymphocyte activation. The objective of this study was to analyze the structural elements that participate in the cellular distribution of Kvβs. It was demonstrated that Kvβ peptides, in addition to the cytoplasmic pattern, targeted the cell surface in the absence of Kv channels. Furthermore, Kvβ2.1, but not Kvβ1.1, targeted lipid raft microdomains in an S-acylation-dependent manner, which was concomitant with peptide localization within the immunological synapse. A pair of C-terminal cysteines (C301/C311) was mostly responsible for the specific palmitoylation of Kvβ2.1. Several insults altered Kvβ2.1 membrane localization. Therefore, growth factor-dependent proliferation enhanced surface targeting, whereas PKC activation impaired lipid raft expression. However, PSD95 stabilized Kvβ2.1 in these domains. This data shed light on the molecular mechanism by which Kvβ2.1 clusters into immunological synapses during leukocyte activation.

**Supplementary Information:**

The online version contains supplementary material available at 10.1007/s00018-022-04269-3.

## Introduction

Voltage-gated potassium channels (Kv) control action potentials in muscles and nerves. Furthermore, Kv plays fundamental functions during leukocyte activation, proliferation and apoptosis. In addition, the association with different accessory proteins further increases their functional diversity. These ancillary interactions affect not only the electrophysiological properties of the channels, but also their localization, trafficking and turnover [1, 2].

The Kvβ family was the first group of proteins identified as Kv channel regulatory subunits. Three different genes (*KCNAB1*, *KCNAB2* and *KCNAB3*) encode this family, including different splicing variants [1]. The crystal structure of the Kv1.2 channel in the presence of Kvβ2.1 confirmed the soluble nature of these Kvβ peptides [3]. These cytoplasmic proteins, via their conserved C-terminal domain, associate with α-subunits by interacting with an N-terminus preserved structure in Kv [4]. In addition to regulating the electrophysiological properties, some Kvβ members alter the trafficking of Kv channels. Therefore, an increase in Kv channels at the cell surface claims chaperone-like effects [5, 6].

While the modulation of the activity of Kv channels by the Kvβ subfamily has been under intensive investigation, the Kvβ subcellular distribution has been poorly analysed. In addition to cytosolic localization, Kvβ1.1, but not Kvβ2.1, associates with the actin-based cytoskeleton [4]. This interaction, placed in the ball-and-chain N-terminal domain of Kvβ, induces resistance to extraction with nonionic detergents [7] and could explain why Kvβ1.1 increases Kv1.1 current inactivation under depolymerization [8, 9]. However, Kvβ2 polarizes in some tissues in association with the microtubular cytoskeleton [10, 11].

From a functional point of view, Kvβ2, lacking the inactivation ball-and-chain domain, enhances folding, glycosylation, trafficking and axonal targeting of Kv1 channels. However, this function is highly dependent on the α-subunit [5, 12]. In addition, Kvβ2 functions as alpha-keto reductase (AKR) [13, 14]. Genetic ablation of Kvβ2 leads to reduced lifespans, occasional seizures, and cold swim-induced tremors. 1p36 deletion syndrome, with a *KCNAB2* genetic deficiency, triggers childhood seizures and altered electroencephalograms [15]. However, a further Kv1 distribution analysis claims that neither the chaperone-like function nor the AKR-like catalytic activity of Kvβ2 would be responsible for this phenotype, pointing to a more complex scenario [16, 17].

Leukocytes express a limited repertoire of Kv channel proteins, including the Kv1.3 channel and Kvβ1 and Kvβ2 regulatory subunits [18, 19]. Similar to Kv1.3, Kvβ peptides are regulated during proliferation and activation in leukocytes. Kv1.3 targets the immunological synapse (IS) during cellular responses. Although there is no direct experimental proof, because ZIP kinases associate with both Kvβ2 and protein kinase C (PKC) ζ, assembling the PKCζ-ZIP-Kv1.3 complexes, evidence most likely situates Kvβ2 within the interactome of the IS [20–22]. ZIP1 and ZIP2 phosphorylate Kvβ2 by PKCζ forming heteromultimeric complexes. In this scenario, PKC activation displaces Kv1.3 from rafts [23], which are concentrated in the IS [24]. Whether Kvβ2 routes to the cluster either by direct association with Kv1.3, ZIP and PKC or by self-targeting is unknown. This information turns crucial to understanding the complex architecture that configures the IS during the immunological response [20, 22, 25].

This study demonstrates, for the first time, the presence of Kvβ regulatory subunits at the plasma membrane. In addition, Kvβ2, but not Kvβ1, targets lipid raft microdomains and concentrates at the immunological synapse. Kvβ2 routes to these spots in the absence of Kv1.3 but is highly dependent on the S-palmitoylation of two distal C-terminal cysteine residues. Furthermore, proliferation and activation differentially altered Kvβ2 localization. While proliferation concentrates Kvβ2 in these domains, PKC-dependent activation decreases its abundance. However, PSD95 stabilizes the presence of this regulatory peptide in rafts.

## Results

### Kvβ localized at the plasma membrane, and Kvβ2.1, but not Kvβ1.1, targeted lipid rafts

**Fig. 1:**
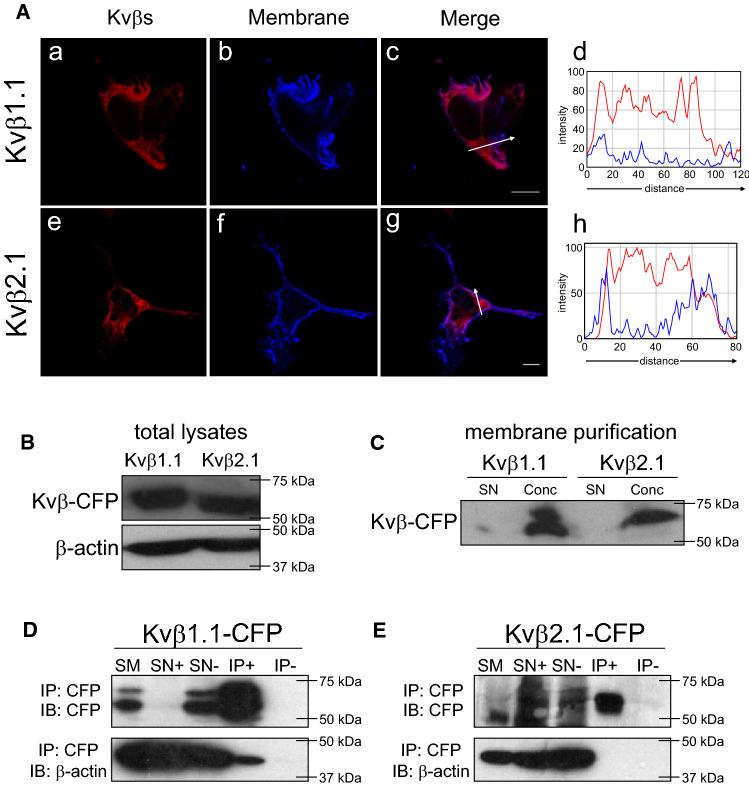
Cytoplasmic Kvβ1.1 and Kvβ2.1 also target the plasma membrane. HEK 293 cells were transfected with Kvβ1.1CFP and Kvβ2.1CFP, and the cellular expression was studied. **A** Kvβ1.1 (a-c) and Kvβ2.1 (e–g) distribution. (Ad and Ah) Pixel-by-pixel analysis of Kvβs and the membrane marker. Histograms derived from arrow sections in c and g. a and e, Kvβ in red; b and f, membrane in blue; c and g, merge; purple indicates colocalization. Scale bars represent 10 µm. **B** Kvβ1.1CFP and Kvβ2.1CFP expression in whole lysates from HEK cells. **C** Purified membranes from HEK cells expressing Kvβ1.1CFP and Kvβ2.1CFP. SN, supernatants; Conc, concentrated membranes. **D**, **E** Coimmunoprecipitation of Kvβ1.1CFP and Kvβ2.1CFP with β-actin. **D** Coimmunoprecipitation of Kvβ1.1CFP with β-actin. **E** Absence of coimmunoprecipitation of Kvβ2.1CFP with β-actin. Top panels: immunoblot (IB) against CFP (Kvβs). Bottom panels: immunoblot (IB) against β-actin. SM, starting material. SN+ , supernatants in presence of antibody. SN-, supernatants in absence of antibody. IP+ , immunoprecipitation in the presence of antibody. IP-, immunoprecipitation in the absence of antibody

The regulatory voltage-gated Kvβ1.1 and Kvβ2.1 peptides are cytosolic proteins that clearly display intracellular phenotypes (Fig. 1Aa–Ah). However, when the Kvβ pattern was analyzed further, pixel-by-pixel analysis revealed some partial colocalization with plasma membrane staining (Fig. 1 Ad and Ah). This Kvβ cell surface expression was further confirmed in enriched plasma membrane preparations (Fig. 1B, C).

Evidence claims that the association of Kvβ1.1, but not Kvβ2.1, with actin filaments would locate Kvβ1.1 near the plasma membrane [7]. Coimmunoprecipitation studies confirmed that, unlike Kvβ2.1, Kvβ1.1 indeed interacted with β-actin (Fig. 1D, E). In addition, the membrane localization of Kvβ subunits was independent of the amount of protein expressed per cell (Fig. S1).

**Fig. 2:**
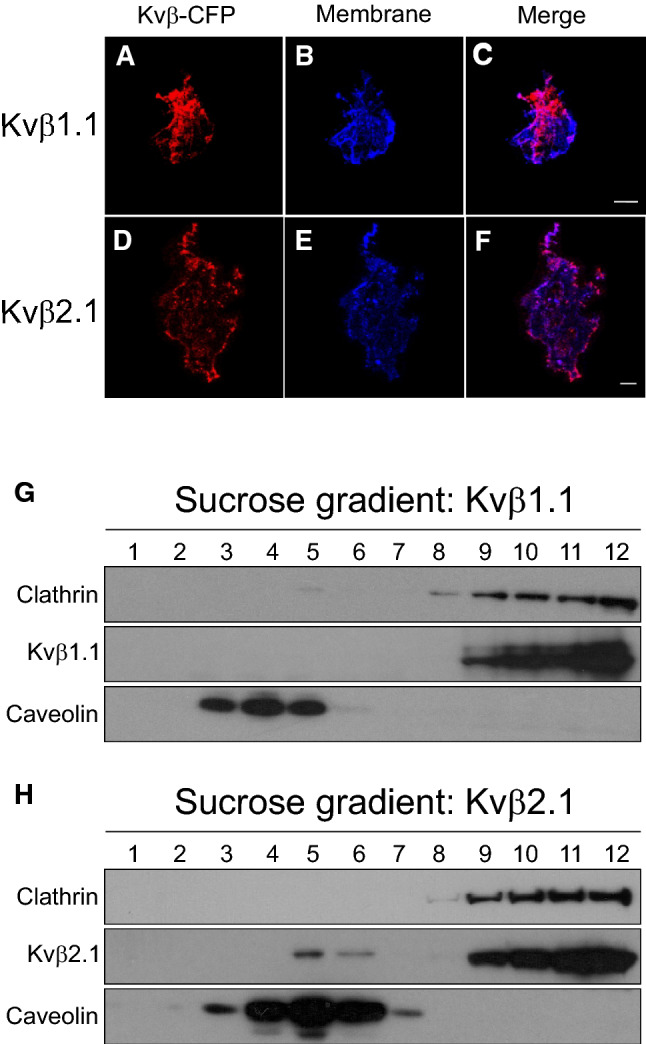
Kvβ expression in cell-unroofing preparations (CUPs) and lipid raft microdomains. **A**–**C** Kvβ1.1CFP and the membrane marker in CUPs. **D**–**F** Kvβ2.1CFP and the membrane marker in CUPs. **A** and **D** Kvβs-CFP in red; **B** and **E** Membrane marker in blue; **C** and **F** merged channel; purple indicates colocalization. Scale bars represent 10 µm. **G** and **H** Lipid raft isolation of HEK cells transfected with Kvβ1.1CFP **G** and Kvβ2.1CFP **H**. Sequential sucrose fractions were extracted from the top (1, lowest density) to the bottom (12, highest density) of the tube and the Kvβ expression analyzed by western blot

To further study the membrane distribution of Kvβ, cell-unroofing preparations (CUPs) were prepared from HEK cells transfected with Kvβ1.1CFP and Kvβ2.1CFP (Fig. 2A–F). Both Kvβ1.1 (Fig. 2A–C) and Kvβ2.1 (Fig. 2D–F) were clearly detected in CUPs, but their distribution differed. While Kvβ1.1 was distributed evenly, Kvβ2.1 mostly appeared as a punctate pattern. Certain punctate patterns of ion channels indicate protein localization in discrete lipid raft microdomains [26]. Because Kvβ2.1 did not interact with β-actin but was present in the plasma membrane, showing a punctate pattern, whether Kvβ2.1 targeted lipid raft microdomains was postulated (Fig. 2H). Interestingly, while Kvβ2.1 localized in *rafts* (8 ± 2%) Kvβ1.1 did not (Fig. 2G, H). Kvβ2.1 lipid raft targeting was further confirmed by cholera toxin β subunit (CTXβ) staining in both whole cells and CUPs (Fig. S2). Several ion channel proteins, including Kv, target lipid rafts by interacting with caveolin via caveolin-binding domains (CBDs) [27, 28]. A structural analysis of Kvβ2.1 revealed several putative CBDs (Fig. S3A). However, coimmunoprecipitation between both Kvβs and caveolin demonstrated that neither Kvβ1.1 nor Kvβ2.1 showed an association with caveolin (Fig. S3B-C). These results suggested that Kvβ2.1 targets the plasma membrane lipid raft microdomains by specific mechanisms independent of actin filaments and not mediated by caveolin interaction.

### S-acetylation drives Kvß2.1 to cell plasma membrane lipid raft microdomains

Soluble proteins may attach to the eukaryotic plasma membrane by lipidic posttranslational modifications such as S-acetylation. Palmitoyl acyltransferases (PATs) add palmitate to cysteine residues, which can be reverted by thiosterases. Palmitoylation helps protein–protein interactions and confers protein stability but also improves trafficking and membrane association [29].

**Fig. 3:**
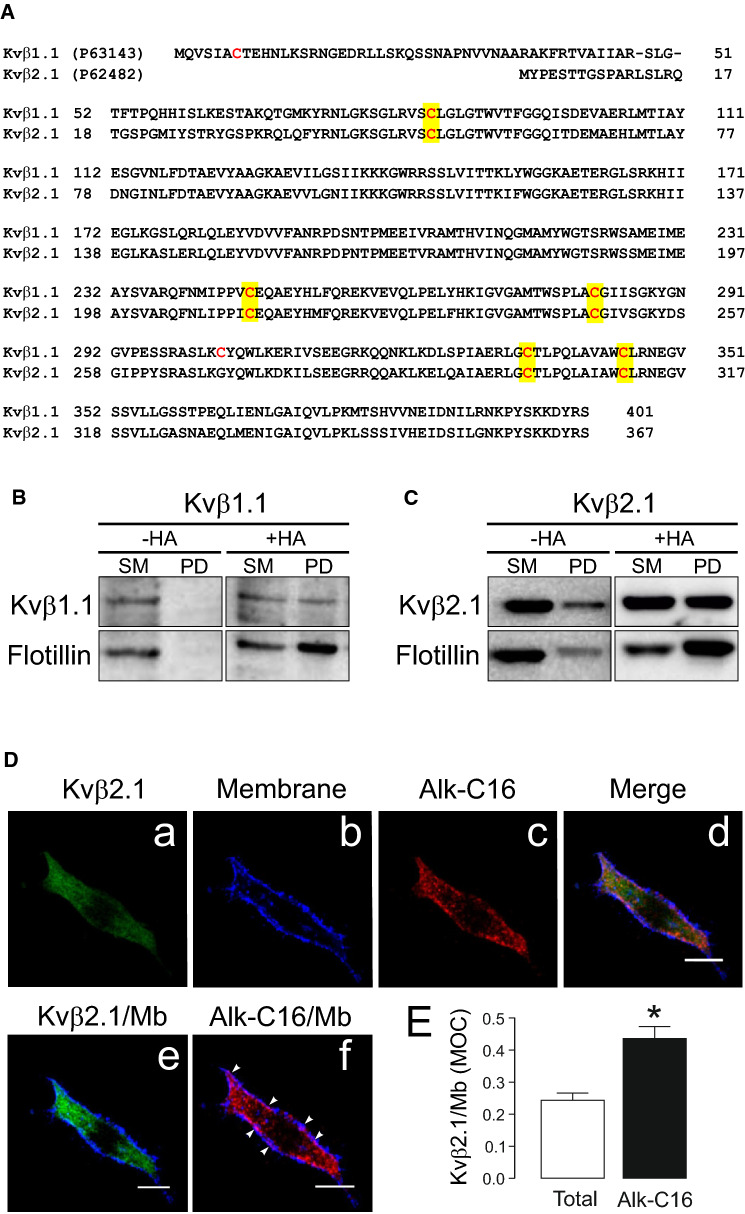
Palmitoylation of Kvβ1.1 and Kvβ2.1. **A** Amino acid sequence alignment of murine Kvβ1.1 and Kvβ2.1. The UniProt (https://www.uniprot.org/) identification number is indicated in brackets. Sequences were analyzed for cysteines. Cysteines are in red. Conserved cysteines are highlighted in yellow. **B**, **C** ABE palmitoilation assay on HEK cells transfected with Kvβ1.1CFP (**B**) and Kvβ2.1CFP (**C**). + HA, presence of hydroxylamine. −HA, absence of hydroxylamine. Top panels, immunoblot against Kvβ1.1 and Kvβ2.1. Bottom panels, immunoblot against flotillin. SM, starting material. PD, pulldown of the palmitoylated proteins. **D** Palmitoylated Kvβ2.1 targets the cell surface. Proximity-ligation-assay (PLA). Palmitic acid 15-hexadecynoic acid was used for Alk-C16 protein palmitoylation. (a) Total Kvβ2.1CFP in green; (b) membrane marker staining in blue; (c) Kvβ2.1CFP Alk-C16 palmitoylation in red; (d) merge panel; (e) colocalization of total Kvβ2.1CFP, in green, with the membrane marker (Mb) in blue; (f) colocalization of Kvβ2.1CFP Alk-C16, in red, with the membrane marker (Mb) in blue. Arrowheads highlight Alk-C16 palmitoylation colocalizing with the cell surface in purple. Scale bars represent 10 µm (**E**) Quantification of membrane (Mb) colocalization with total and palmiyoylated (Alk-C16) Kvβ2.1 using Mander’s overlap coefficient (MOC). *p < 0.05 (Student’s *t* test) vs. total Kvβ2.1. Values are mean ± SE of 30 cells

Evidence situates Kvβ2.1 at the lymphocyte IS, which is enriched in lipid rafts [20, 24]. Because Kvβ2.1 targeted raft microdomains by actin- and caveolin-independent mechanisms, we analyzed whether Kvβ2.1 could undergo palmitoylation, facilitating membrane targeting. The mKvβ1.1 and mKvβ2.1 structures (https://www.UniProt.org) share five cysteine putative targets for S-acylation (Fig. 3A). Palmitoylation ABE analysis of Kvβ2.1 and Kvβ1.1 showed that both peptides were palmitoylated (Fig. 3B, C). Thus, biotin pulled down (PD) samples, in the presence of hydroxylamine (+ HA), which cleaves palmitoyl-cysteine thioester linkages, revealed the palmitoylation of flotillin (positive control, which also targets rafts) and Kvβ2.1, as well as Kvβ1.1 (Fig. 3B, C). A further PLA assay definitely demonstrated that cell surface Kvβ2.1 was indeed S-palmitoylated in HEK 293 cells (Fig. 3D). In fact, membrane colocalization analysis indicated that palmitoylated Kvβ2.1 (Alk-C16) targeted the plasma membrane better than total Kvβ2.1 (Fig. 3E).

**Fig. 4:**
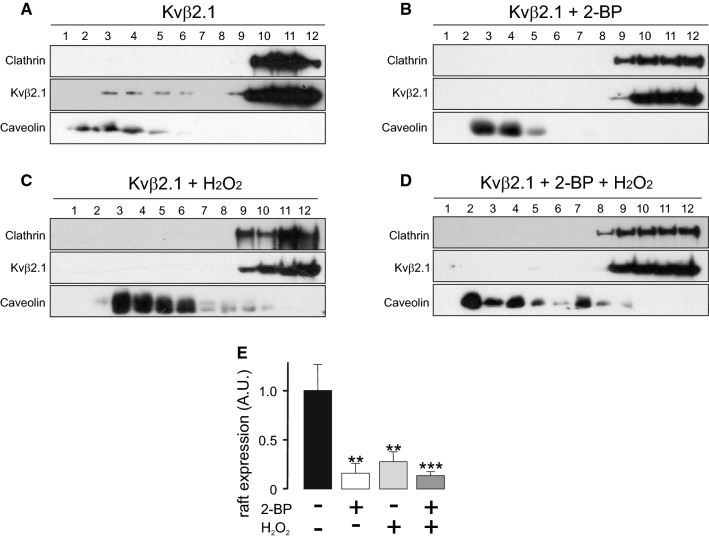
Palmitoylation-dependent lipid raft targeting of Kvβ2.1. **A**–**D** Lipid raft microdomains were isolated in the absence (**A**) or presence of (**B**) 2-bromopalmitate (2-BP), (**C**) H_2_O_2_ or both (**D**), and lipid raft fractions were analyzed for Kvβ2.1CFP by western blot. Lipid raft fractions were sequentially extracted from the top (1, lowest density and highest buoyancy) to the bottom (12, highest density and lowest buoyancy) of the tube. (**E**) Quantification of the expression of Kvβ2.1CFP in low-density fractions, defined by the presence of caveolin, relativized by the total amount of expression. A.U., arbitrary units. **p < 0.01; ***p < 0.001 vs. no additions (Student’s *t* test)

The S-acylation of Kvβ2.1 could have no correlation with lipid raft targeting because Kvβ1.1 was also palmitoylated. Therefore, two additional treatments approached lipid raft targeting: 2-bromopalmitate (2-BP) and H_2_O_2_ (Fig. 4A–C). 2-BP competes against palmitate for the palmitoyltransferase domain of PATs. In addition, H_2_O_2_, a strong oxidant, impairs palmitoylation on the SH groups by the formation of cistine bonds [30, 31]. Additionally, the Kvβ family exhibits NADPH oxidoreductase activity, which buffers oxidant reactions. Therefore, by combining both treatments, the presence of Kvβ2.1 in lipid raft domains related to either palmitoylation or oxidation could be analyzed (Fig. 4D). Around 10% of Kvβ2.1 targeted to lipid rafts, but this was impaired by 2-BP (Fig. 4A, B) and H_2_O_2_ (Fig. 4C) by approximately 83.9% ± 9.8 and 72.3% ± 10, respectively (Fig. 4E). The combination of both treatments (Fig. 4D) triggered a similar reduction (86.6% ± 3.9). These results pointed to S-palmitoylation as responsible for the lipid raft targeting of Kvβ2.1 independent of oxidoreductase activity.

### Human T lymphocytes concentrate palmitoylated Kvß2.1 at the immunological synapse

**Fig. 5:**
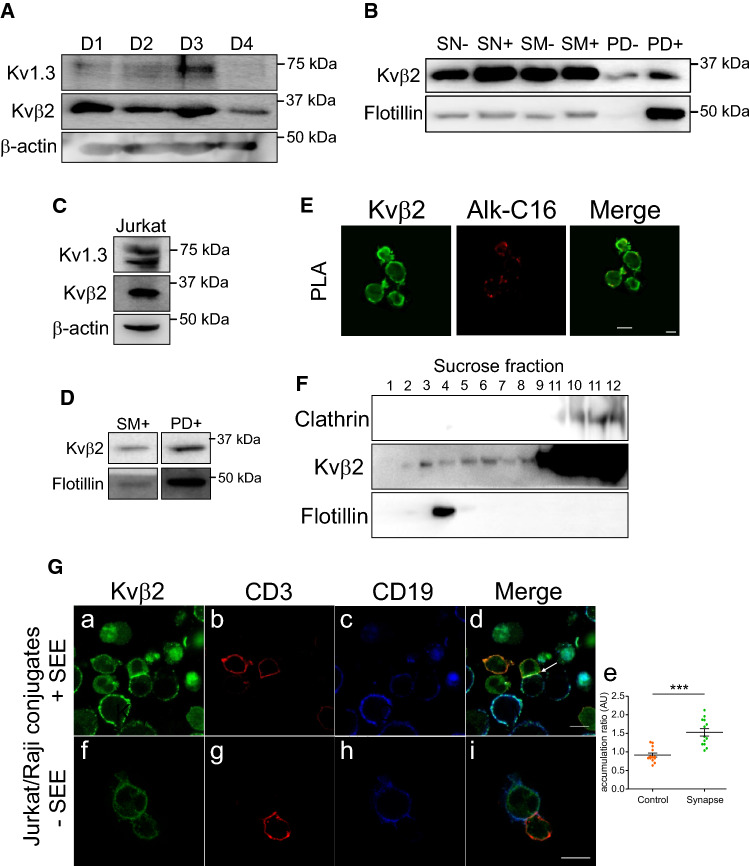
Human T lymphocytes express palmitoylated Kvβ2.1, which targets lipid raft microdomains and concentrates in the IS during the immunological response. (**A**) Human CD4 + lymphocytes express Kvβ2 and Kv1.3. T lymphocytes from 4 different donors (D1-4) were obtained and analyzed. (**B**) Kvβ2 undergoes palmitoylation in human CD4 + T-cells. SN, supernatant in the absence (−) or the presence (+) of HA. SM, starting material in the absence (−) or the presence (+) of HA. PD, pulldown of palmitoylated proteins in the absence (−) or presence (+) of HA. **C** Human Jurkat T lymphocytes express Kvβ2 and Kv1.3. **D** Kvβ2 undergoes palmitoylation in human Jurkat T cells. SM, starting material in the presence of HA. PD, pulldown of palmitoylated proteins in the presence of HA. **E** Proximity ligation assay (PLA) in Jurkat lymphocytes. Palmitic Alk-C16 Kvβ2 palmitoylation. Total Kvβ2 in green; Alk-C16 Kvβ2 palmitoylation in red; merged panel highlights Alk-C16 palmitoylation at the cell surface. **F** Kvβ2 targets lipid rafts in Jukat cells. Lipid raft fractions were sequentially extracted from the top (1, lowest density and highest buoyancy) to the bottom (12, highest density and lowest buoyancy) of the tube. Because T cells lack the expression of caveolin, flotillin identified lipid rafts. **G** Cell conjugates between human Jurkat T cells and human Raji B lymphocytes. (Ga-Ge) SEE-activated B lymphocytes were cocultured in the presence of Jurkat cells. Merged panel showing triple colocalization in white (white arrow) localizes Kvβ2 in the IS. Panel Ge shows the accumulation ratio of Kvβ2 at the IS vs. the entire Kvβ2 cell intensity. Values represent mean ± SE. ***p < 0.01 Student’s *t* test. (Gf-Gi) Non-SEE- activated B lymphocytes were cocultured in the presence of Jurkat cells. Note that neither Kvβ2 accumulation nor IS formation is observed. Cells were stained against Kvβ2 (green), CD3 (marker of T cells, red), and CD19 (marker of B cells, blue). Bars are 20 μm

We showed that palmitoylated Kvβ2.1 targeted plasma membrane lipid raft microdomains. In addition, Kvβ2.1 interacts with ZIP1/2 and PKC, providing an effective phosphorylation cluster for signaling in the IS [20, 22, 25]. During T-cell activation, IS, enriched in lipid rafts, concentrates Kv1.3 channels, which in turn may associate with Kvβ2.1 to fine tune the immune response [32]. However, our data would indicate that Kvβ2.1 is located in IS via palmitoylation, even in the absence of Kv1.3. Therefore, the AKR activity of Kvβ2.1 in addition to further functions would be independent of channel expression. In this scenario, evidence claims Kvβ2.1 localization at IS [20]. Although palmitome studies identify Kvβ2 in brain [33, 34], this protein is not found in some leukocyte analysis [35–39]. Human CD4 + lymphocytes and human Jurkat T cells expressed Kvβ2 (Fig. 5A, C). Biotin pulled down extracts (PD), in the presence of hydroxylamine, revealed that CD4 + lymphocytes (Fig. 5B) and Jurkat cells (Fig. 5D) expressed palmitoylated Kvβ2.1, which was located on the plasma membrane surface (Fig. 5E) and targeted lipid rafts (Fig. 5F) in Jurkat lymphocytes. Cell conjugates between SEE-activated human B lymphocytes (Raji) and human T lymphocytes (Jurkat) demonstrated that while Kvβ2.1 evenly stained the surface of T cells, in the absence of IS, Kvβ2.1 concentrated at the IS once the synapse was achieved (Fig. 5Ga–e). Non-SEE activated Raji cells generate no synapse with Jurkat T cells and Kvβ2 did not accumulate in cell-to-cell contact surfaces (Fig. 5Gf–i).

### Distal C-terminal cysteines are responsible for the specific palmitoylation of Kvß2.1

**Fig. 6:**
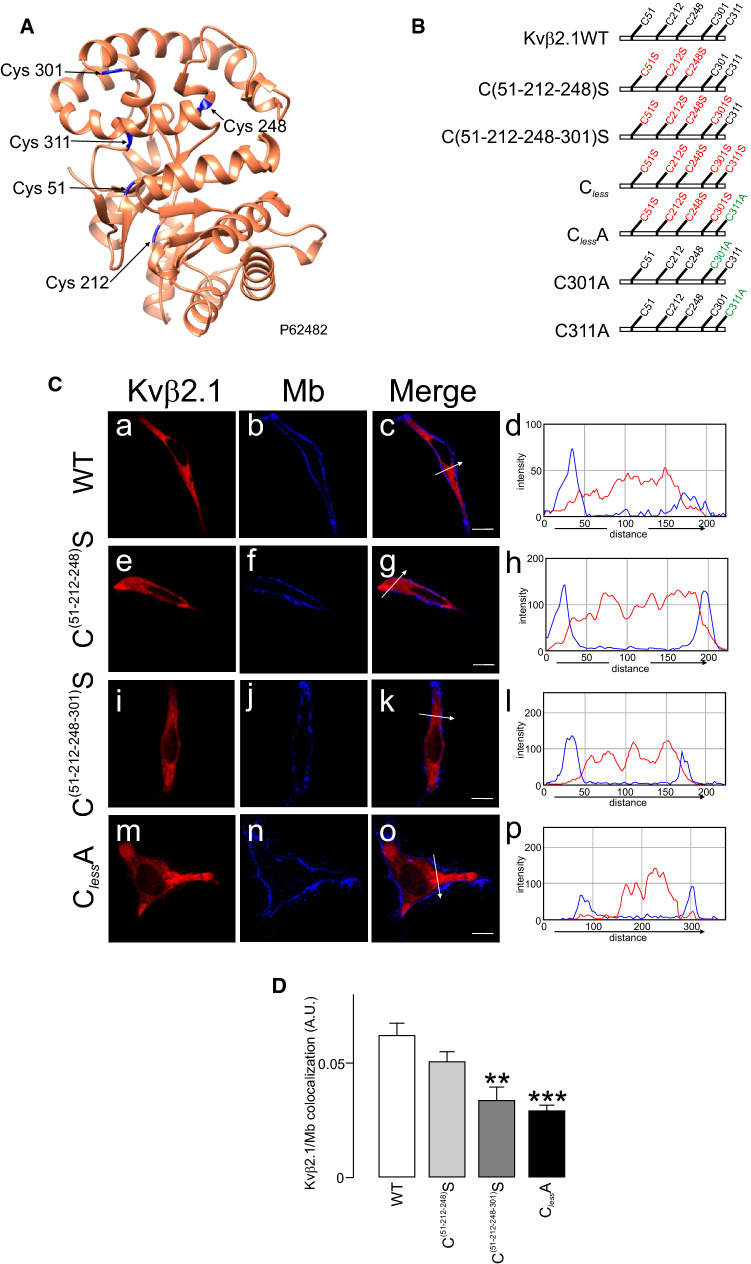
Cellular distribution of different Kvβ2.1 mutants. **A** Cartoon of the crystal structure of murine Kvβ2.1 (UniProtKB—P62482), highlighting 5 cysteines in blue. **B** Representative cartoon of Kvβ2.1 mutants. While Ser substitutions are indicated in red, Ala mutations are indicated in green. **C** Cellular staining of Kvβ2.1 WT (a-d), C^(52–212−248)^S (e–h), C^(52–212−248–301)^S (i-l) and Kvβ2.1C_less_A (m-p). Panels d, h, l and p show the pixel-by-pixel analysis of white arrow sections in c, g, k and o, respectively. Scale bars represent 10 µm. **D** Quantification of membrane colocalization using Mander’s overlap coefficient (MOC). **p < 0.01; ***p < 0.001 (Student’s *t* test) vs. WT. Values are mean ± SE of 20–30 cells

We demonstrated that palmitoylated Kvβ2.1 reached plasma membrane lipid raft microdomains and polarized into IS during the immune response. Because 2-BP is promiscuous and targets PAT enzymes, transporters and many palmitoylated proteins [40], 2-BP’s impairment of Kvβ2’s targeting to lipid rafts (Fig. 4) must be taken with caution. Therefore, the molecular determinants involved were further analyzed. The five S-acylated putative Cys residues in Kvβ2.1 (51, 212, 248, 301 and 311) were mutated to Ser, maintaining structural similarity (Fig. 6A, B). Surprisingly, while C(51–212-248–301)S preserved the regular intracellular pattern of Kvβ2.1, with limited cell surface localization, the introduction of the C311S mutation in combination with the rest of cysteine (C_less_) triggered anomalous plasma membrane distributions (Fig. S4). This altered behavior could be the consequence of the introduction of an artificial PKC phosphorylation site (Fig. S4B). Therefore, this substitution was discarded, and new Kvβ2.1 C301A, C311A and C_less_A mutants were generated, which conserved the WT phenotype (Fig. 6C). The sequential substitution of cysteines steadily triggered the loss of membrane localization, which was pronounced when the distal C-term Cys (301 and 311) were mutated (C51-212-248-301S; C_less_A) (Fig. 6D).

**Fig. 7:**
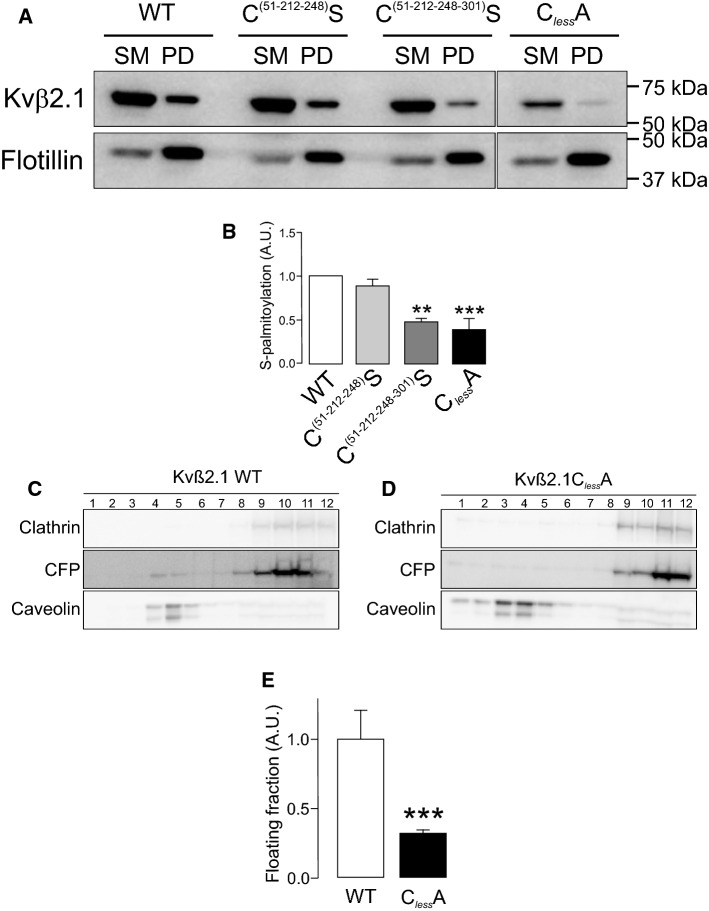
Palmitoylation of Kvβ2.1 WT and mutants. HEK 293 cells were transfected with Kvβ2.1CFP WT and several cysteine mutants (C^(51–212−248)^S, C^(51–212−248–301)^S and C_less_A), and palmitoylation was analyzed. **A** Representative ABE experiment of Kvβ2.1 WT and C^(51–212−248)^S, C^(51–212−248–301)^S and C_less_A mutants. Flotillin was the positive control. SM, starting material; PD, palmitoylated pull down. **B** Quantification of pulldowns normalized to starting materials. Values are mean ± SE of three independent experiments. **p < 0.01; ***p < 0.001 (Student’s *t* test) vs. WT. White bar, Kvβ2.1 WT; light gray bar, C^(51–212−248)^S; dark gray bar, C^(51–212−248–301)^S; black bar, Kvβ2.1C_*less*_A. **C**, **D** Localization of Kvβ2.1 (**C**) and Kvβ2.1 C_less_A (**D**) in lipid rafts. Lipid raft fractions were sequentially extracted from the top (1, low density and high buoyancy) to the bottom (12, high density and low buoyancy) of the tube. (**E**) Quantification of the abundance of Kvβ2.1CFP in low buoyant fractions, defined by caveolin, relativized by the total amount of expression. ***p < 0.001 (Student’s *t* test) vs. WT. Values are mean ± SE of three independent experiments. White bar, Kvβ2.1 WT; black bar, Kvβ2.1C_less_A

Whether S-palmitoylation was concomitant with the cell surface expression of Kvβ2.1 was further investigated. The ABE data showed that only when Cys 301 and 311 were individually substituted (C301A, C311A) or combined with the rest of Cys (C51-212–248-301S; C_less_A) the Kvβ2.1 palmitoylation was clearly impaired (Fig. 7A, B; Fig. S5). Therefore, evidence demonstrated that the C301 and C311 C-terminal distal residues were the main targets for PATs in Kvβ2.1. Furthermore, palmitoylation-dependent lipid raft and plasma membrane targeting was further confirmed. Thus, unlike Kvβ2.1 WT, Kvβ2.1 C_less_A neither targeted to rafts (Fig. 7C–E) nor to cell surface (Fig. S6).

### Proliferation and activation differentially alter Kvβ2.1 localization

**Fig. 8:**
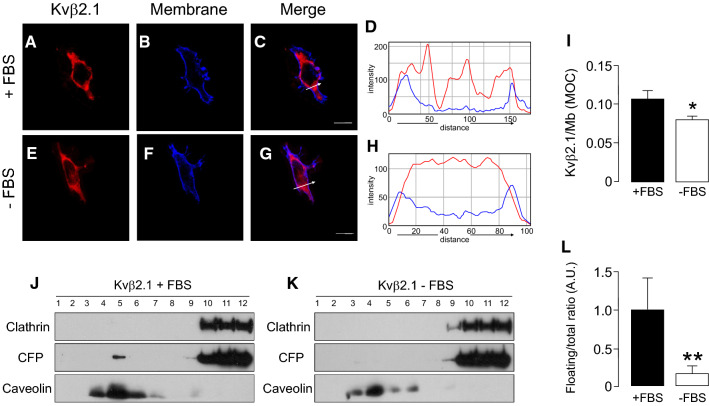
FBS-dependent proliferation increases Kvβ2.1 membrane and lipid raft targeting. HEK 293 cells were transfected with Kvβ2.1CFP. After 24 h of FBS deprivation, cells were further cultured for 24 h in the presence (**A**–**D**) or absence (**E**–**H**) of 10% FBS, and the cellular distribution of Kvβ2.1 was analyzed. **A**, **E** Kvβ2.1 CFP in red. **B**, **F** WGA membrane staining in blue. **C**, **G** merge, purple indicates colocalization. Scale bars represent 10 µm. **D** and **H** Pixel-by-pixel analysis of the arrow sections in **C** and **G**, respectively. **I** Quantification of membrane targeting using Mander’s overlap coefficient (MOC). *p < 0.05 vs. + FBS (Student’s *t* test). Values are mean of 20–30 cells. **J**, **K** Lipid raft localization of Kvβ2.1CFP in the presence (**J**) or absence (**K**) of FBS. (**L**) Quantification of Kvβ2.1CFP floatability. Expression of Kvβ2.1CFP in low buoyant fractions (defined by caveolin expression) relative to the total amount. **p < 0.01 vs. + FBS (Student’s *t* test). Values are mean of 4 independent experiments. Black bars, presence of FBS; white bars, absence of FBS

Lipid rafts are membrane platforms that initiate cell signaling. These microdomains reduce the spatial distance between signaling proteins and their targets [41]. Considering Kvβ oxidoreductase function and Kv modulation, the positioning of Kvβ2.1 in lipid raft microdomains within the IS was highly relevant. Because Kvβ subunits exhibit differential regulation upon proliferation or activation in macrophages [18], it was wondered whether targeting lipid rafts, which was dependent on S-palmitoylation, underwent regulation. Therefore, HEK cells were cultured in the absence of FBS and analyzed Kvβ2.1 spatial location. In the absence of growth factors (-FBS), the plasma membrane Kvβ2.1 localization slightly decreased (Fig. 8A–I) concomitant with a notable disappearance from floating fractions (Fig. 8J–L).

**Fig. 9:**
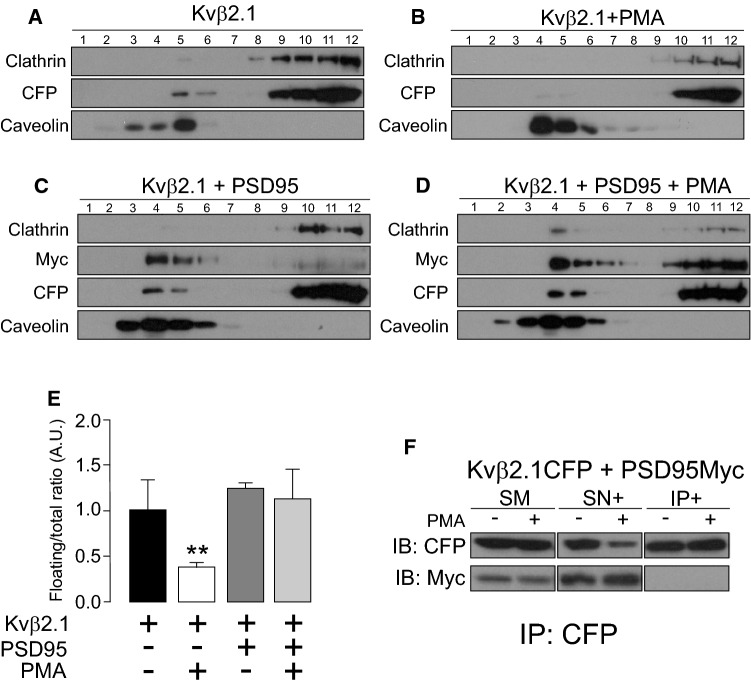
Lipid raft microdomain expression of Kvβ2.1 in the presence of PMA_._ HEK 293 cells were transfected with Kvβ2.1CFP. Isolation of lipid rafts was performed after 1 μM PMA incubation for 30 min at 37 °C. **A** Kvβ2.1CFP expression in lipid raft fractions in the absence of PMA. **B** Kvβ2.1CFP expression in the presence of PMA. **C**, **D** HEK 293 cells were cotransfected with Kvβ2.1CFP and Myc-PSD95 in the absence (**C**) or presence (**D**) of PMA. Immunoblot against CFP shows Kvβ2.1CFP. Immunoblot against Myc indicates Myc-PSD95. **E** Quantification of the Kvβ2.1 floatability, indicated by caveolin expression, relativized to the total amount of expression. **p < 0.01 vs. Kvβ2.1 in the absence of PMA (Student’s *t* test). Black bar, Kvβ2.1 alone; white bar, Kvβ2.1 in the presence of PMA; dark gray bar, Kvβ2.1 in the presence of PSD95 but in the absence of PMA; light gray bar, Kvβ2.1 in the presence of PSD95 and PMA. Values are men ± SE of 4 independent experiments. **F** Kvβ2.1 does not interact with PSD95. Cells were cotransfected with Kvβ2.1CFP and Myc-PSD95 in the absence (−) or presence (+) of PMA. Total lysates were coimmunoprecipitated against Kvβ2.1CFP (IP: CFP) and immunoblotted (IB) against CFP (Kvβ2.1) and myc (PSD95). SM, starting material; SN + : supernatant in the presence of antibody. IP + : Immunoprecipitation in the presence of the antibody

Evidence demonstrates that PKC initiates signaling events within IS [21]. Previous works from the authors’ laboratory report a PMA-dependent exit of Kv1.3 from raft domains prior to endocytosis of the channel [23]. Because Kvβ2.1, which is phosphorylated by PKC [42], was present at lipid rafts, it was analyzed whether PMA would also regulate the location of this auxiliary subunit. Therefore, lipid raft microdomains were isolated in the absence or presence of PMA, and Kvβ2.1 expression was analyzed (Fig. 9 A-D). PMA reduced the distribution of Kvβ2.1 to nonfloating fractions by 67.4% ± 4.3 (Fig. 9E).

MAGUK proteins participate in IS formation, NFAT activation, cytokine secretion and negatively regulate lymphocyte proliferation. hDgl1 (synapse-associated protein 97, SAP97) is located at the IS, and hDgl4 (postsynaptic density protein 95, PSD95) is required for uropod formation in T-cells [43, 44]. Furthermore, PSD95, interacting with Kv1.3 at the IS, stabilizes the channel in rafts upon PKC-dependent endocytosis. Palmitoylated PSD95 targets lipid rafts interacting with PKCα [45–47]. Taking all this in mind, the effect of PSD95 on Kvβ2.1 were analyzed upon PKC-dependent activation. The data indicated that PSD95 stabilized Kvβ2.1 into floating fractions, preventing the PKC-dependent lipid raft displacement observed with PMA (Fig. 9C–E). However, unlike Kv1.3 [23], PSD95 did not interact with Kvβ2.1 (Fig. 9F). Therefore, these results indicated that PSD95 functions on Kvβ2.1 by an indirect mechanism stabilizing lipid rafts buffering PKC effects.

PKC activation triggers Kv1.3 endocytosis, increasing endosomal localization and lysosomal degradation of the channel, which is crucial for Kv1.3-related cell physiology [23]. Although mostly intracellular, Kvβ2.1 shares Kv1.3 spatial distribution and PKC-dependent regulation. Therefore, it was wondered whether PKC activation mediates a similar regulatory mechanism on Kvβ2.1. However, PMA-dependent PKC activation neither increased the presence of Kvβ2.1 in early endosomes nor significantly augmented its ubiquitination (Fig. S7A–D). Furthermore, unlike Kv1.3 (Fig. S7E), the stability of Kvβ2.1 was not altered by PKC-dependent activation. Thus, even after 24 h of PMA incubation neither lysosomal (BA, bafilomycin) nor proteasome MG (MG132) functions altered Kvβ2.1 abundance (Fig. S7 F,G).

**Fig. 10:**
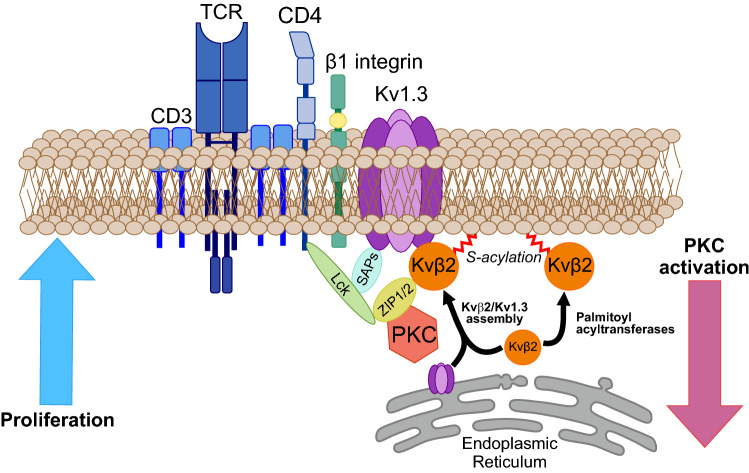
Cartoon representing the structural Kv1.3-associated proteins in the T-lymphocyte immunological synapse. The Kv1.3 channelosome merges a number of proteins modulating channel function at the IS during immunological synapses. PSD95 (also named synapse-associated protein 90; SAP90), which is encoded by the *hDLG4* (discs large homolog 4) gene, stabilizes Kv1.3 at the IS. SAP97 (synapse-associated protein 97; *hDlg1*) plays similar roles. Therefore, SAP peptides bind to the PDZ domain in the C-terminus of Kv1.3, coupling p56lck to CD4. Kvβ2 links the N-terminus of the Kv1.3 channel to the ZIP1/2 protein, which may interact with several partners, such as p56lck and PKC. CD3 and Kv1.3 are in molecular proximity, and the channel interacts with β1-integrins. Our data indicate that ~ 10% of Kvβ2 targets to lipid rafts either associated or not with Kv1.3 and situate palmitoylated Kvβ2 (red sparkline) at the IS, independent of the Kv1.3 interaction, and stabilized by PSD95. Kvβ2 may link cellular metabolic activity and redox state with calcium signaling in lymphocytes. Kvβ2 also serves as a bridge with ZIP-1/2, which also links the complex to p56lck. Other proteins within IS are the T-cell receptor (TCR), CD3 and CD4 accessory proteins. Kvβ2 in activated T cells concentrates on the IS during synapse formation. Under proliferation, Kvβ2 targets lipid rafts, which are concentrated at the IS. In contrast, PKC activation triggers a lipid raft displacement of Kvβ2, which PSD95 counteracts

Our results indicated that Kvβ2.1, even in the absence of Kv1.3, targets the IS by a palmitoylation-dependent mechanism. S-acylation may also play a role in the functional coupling of Kvβ2 and Kv1.3, as has been for BK channel alpha and beta subunits [48], and such role will be discerned in future studies. The information points to the intrinsic importance of Kvβ2.1 function, independent of Kv1.3, during the immune response. In addition, the data show that palmitoylated Kvβ2.1 targets the lipid raft-enriched IS (Fig. 10). In fact, a signaling complex consisting of CD4, Kv1.3, Kvβ2, SAP97 (hDlg1) and ZIP clusters at the IS in human T cells [20, 22, 25]. In this scenario, PSD95 (also known as SAP90 from the synapse-associated protein—SAP—family), which stabilizes Kv1.3 at rafts, would share effects on Kvβ2.1. However, unlike Kv1.3, the mechanism would not involve direct association. Furthermore, while proliferation increased the amount of Kvβ2.1 in lipid rafts, PKC-dependent activation drove the peptide outside rafts, without compromising the peptide stability (Fig. 10).

## Discussion

Voltage-dependent K^+^ channels control the resting potential of mammalian cells. In addition, Kv participate in a myriad of physiological functions. To achieve that role, Kv associate with a number of ancillary proteins that further increase their function. The Kvβ regulatory subunit family is the most important group of peptides that, associating with many Kv channels, provide diversity [1]. We found that cytoplasmic Kvβ2.1 was located at the cell surface and, unlike Kvβ1.1, partially targeted lipid rafts in the absence of Kv subunits. This spatial localization, which situates Kvβ2.1 in the IS during the immune response, was mediated by specific S-acylation in two distal C-terminal cysteines (C301 and C311), which are far from the NADP^+^ binding pocket. S-palmitoylation is related to lipid raft localization, and soluble proteins may route to the cell surface via this lipidation [49]. Therefore, the results of this study are in line with those of palmitoylated SNAP-25, which routes to the plasma membrane, contributing to the formation of the SNARE complex [50]. Furthermore, the palmitoylation of the DLK kinase, critical for axon-to-soma retrograde signaling upon nerve injury, locates DLK in trafficking vesicles [51].

Kvβ1.1 and Kvβ2.1 are palmitoylated, but only the last targeted to lipid rafts. Although this location may be intriguing for a cytoplasmic protein, evidence situates this peptide in the IS complex in lymphocytes at the onset of the immunological response. Several palmitoyl acyltransferases, such as DHHC18 or DHHC13, are expressed in lymphocytes and may underlie the palmitoylation of Kvβs [35–39]. The data demonstrated that S-acylation controls the Kvβ2.1 location and concentrates this peptide in this location upon activation in cell conjugates. In addition, the differential lipid raft targeting of Kvβ2.1 by FBS-dependent proliferation or PMA-induced activation suggests that the abundance of Kvβ2.1 in these domains might serve to differentially regulate cell functions. Evidence demonstrates that Kvβ2.1 modulates the Kv1 and Kv4 families [52, 53]. Upon specific combinations of Kvβ2.1/Kv channels, the half-voltage activation switches to more negative values [54]. Although Kvβ2.1 does not contain the ball-and-chain inactivation system, this protein enhances the surface expression of some channels. Therefore, a possible chaperone-like function for Kvβ2.1 was claimed [6]. However, little is known about the role of this protein in the absence of pore-forming subunits. Computational analysis showed that Kvβ2.1 forms part of the AKR family, and several studies have demonstrated that it is an active enzyme with endogenous substrates [55]. These results suggest putative effects of Kvβ2.1-AKR activity on Kv currents. However, while the interaction with NADPH was required, most likely enhancing the proper folding of Kvβ2.1, the impairment of the enzymatic activity has no major changes on Kvβ2.1 function [56, 57].

Considering that Kvβ2.1 underwent palmitoylation in the absence of α-subunits and that AKR activity is not necessarily coupled to control Kv channels, Kvβ2.1 could act—independent of Kv channels—as a sensor for the redox state of the cell. It was demonstrated that Kvβ1.1 does not target lipid rafts but is placed at the plasma membrane via actin interaction [7]. In this context, Kvβ1.3, closer to Kvβ1.1 than Kvβ2.1, exhibits a slower hydride transfer than Kvβ2.1 [58]. Therefore, the cell could place redox detectors with a specific sensing rate at the plasma membrane. Concomitantly, Kvβ2.1 can preferably reduce aldehydes and ketones of membrane-derived oxidized lipids, highlighting the importance of the Kvβ membrane location [13, 14].

As mentioned above, Kvβ2.1 lipid raft localization was controlled by different insults. Upon proliferation, Kvβ2.1 increased its colocalization in lipid rafts. However, PMA impairs Kvβ2.1 presence in these subcellular domains and, similar to Kv1.3, this was partially counteracted by PSD95. The effect of PSD95 could involve an interaction with PKC rather than associating with Kvβ2.1. Therefore, Kvβ2.1 functions as a redox sensor at IS during the immune response, and MAGUK proteins stabilize the architecture of these multimeric domains. In these spots, several proteins interact with Kvβ2.1 [20, 22]. For instance, ZIP1, ZIP2 and ZIP3 link this protein to PKCζ, which also supports the IS location. These partners may further interact with PKA, EB1, KIF3/kinesin II, TRPV1 and TREK, among others, triggering a complex scenario [11, 21, 59–61]. Because Kvβ2.1 places in lipid rafts following palmitoylation, its partners may be translocated to those domains. This idea suggests that the Kvβ family, in the absence of Kv channels, could exert functions of signaling platforms. Kvβ2.1 interacts with TRPV1, augmenting channel trafficking to the plasma membrane [60]. This balance could be even more complex because other ion channel ancillary subunits, such as Navβ1, CaM, caveolin and KCNE4, modulate Kv1.3 [62–64]. In this context, the control of Kvβ2.1 distribution by FBS and PKC activation would fine-tune the Kv1.3 channelosome architecture. Further experiments need to be performed to decipher the redox control of these multicomplexes.

Evidence highlights the idea that some functions of Kvβ2.1 have yet to be discovered. Kvβ2.1 null mice exhibit mild effects compared with severe consequences by annihilating Kv1.1 and Kv1.2 at the nervous system [16]. However, Kvβ2.1 association with Kv channels triggers sensitivity to hypoxia either by direct (Kv4.3) or indirect (Kv2.1) associations [65]. Furthermore, the fact that Kvβ2.1 AKR activity does not exert major changes in the control of Kv channels, suggesting that Kvβ2.1 could function as either a moonlighting protein or a scaffolding hub [56]. The NADP^+^ binding ability of Kvβ subunits is required for cell surface trafficking of Kv1 channels in mammalian cells as well as axonal targeting [10, 56]. The fact that palmitoylated Kvβ2 concentrates in IS during the immune response, also associating to many partners such as Kv1.3, ZIP1/2 and PKC in these microdomains, suggests an exciting yet deciphered physiological role.

## Materials and methods

### Expression plasmids and site-directed mutagenesis

mKvβ1.1 and mKvβ2.1 were provided by M.M. Tamkun (Colorado State University). mKvβ1.1 and mKvβ2.1 were subcloned into pECFP-N1 (Clontech). mKvβ2.1CFP mutants were generated using QuikChange lightning multisite-directed mutagenesis kits (Stratagene). All constructs and mutants were verified using automated DNA sequencing. Myc-PSD95 was a generous gift from Dr. F. Zafra (Centro de Biología Molecular Severo Ochoa, Madrid).

### Cell culture, transfections and pharmacological treatments

HEK-293 cells were cultured on DMEM (LONZA) containing 10% fetal bovine serum (FBS) supplemented with penicillin (10.000 U/ml), streptomycin (100 μg/ml) and 4 mM l-glutamine (Gibco). Human Jurkat T lymphocytes and Raji B cells were cultured in RPMI 1640 medium (Life Technologies) supplemented with 10% FBS and antibiotics. In some experiments, human CD4 + T cell subsets were used. Cells were isolated from peripheral whole blood using a negative selection Rosette Sep^™^ kit from STEMCELL^™^ Technologies. Human T lymphocytes were cultured at 37 °C and 5% CO_2_ in RPMI 1640 medium (Life Technologies) supplemented with 10% FCS, 1% glutamine, 1% penicillin–streptomycin (Gibco), 1 × nonessential amino acid solution (Thermo Fisher Scientific), 10 mM HEPES (Life Technologies) and 50 U/ml IL-2 (Bionova). To generate T cell blasts, the Dynabeads Human T-Activator CD3/CD28 for T cell expansion and activation kit (Life Technologies) was used following the manufacturer’s instructions. Human T cell blasts were used after 6–7 days of the expansion protocol. No IL-2 was supplemented in media the day before an experiment.

For confocal imaging and coimmunoprecipitation experiments, cells were seeded (70–80% confluence) in six-well dishes containing polylysine-coated coverslips or 100 mm dishes 24 h before transfection. Lipotransfectin^®^ (Attendbio Research) was used for transfection according to the supplier’s instructions. The amount of transfected DNA was 4 µg for a 100 mm dish and 500 ng/well of a six-well dish (for coverslip use). Next, 4–6 h after transfection, the mixture was removed from the dishes and replaced with new fresh culture media. All experiments were performed 24 h after transfection.

In some experiments, 24 h transfected HEK cells were incubated with 1 µM PMA at 37 °C for indicated times. For 2-bromopalmitate treatment, HEK-transfected cells were starved for 3 h with DMEM supplemented with 1% dialyzed FBS, and 60 nM 2-bromopalmitate was added for 3 more hours at 37 °C. H_2_O_2_ (400 µM) was added for 30 min at 37 °C.

### Protein extraction, membrane-enriched preparations, coimmunoprecipitation and western blotting

Cells were washed twice in cold PBS (phosphate-buffered saline; 137 mM NaCl, 2.7 mM KCl, 8 mM Na_2_HPO_4_, and 2 mM KH_2_PO_4_, pH 7.4) and lysed on ice with lysis buffer (5 mM HEPES, 150 mM NaCl, 1% Triton X-100, pH 7.5) supplemented with 1 μg/ml aprotinin, 1 μg/ml leupeptin, 1 μg/ml pepstatin and 1 mM phenylmethylsulfonyl fluoride as protease inhibitors. Lysates were incubated for 20 min at 4 °C and further spun for 20 min at 14,000 rpm. The protein content of the supernatant was determined using the Bio–Rad Protein Assay (Bio–Rad).

Membrane-enriched preparations were purified from 24 h transfected HEK 293 cells. Cells were washed twice in cold PBS and scrapped in 1 mL of HB buffer (20 mM HEPES pH 7.4, 1 mM EDTA, 255 mM sucrose) supplemented with 1 μg/ml aprotinin, 1 μg/ml leupeptin, 1 μg/ml pepstatin and 1 mM phenylmethylsulfonyl fluoride as protease inhibitors. Samples were passed through a 25-gauge needle ten times, and lysates were centrifuged for 5 min at 3000 × *g* and 4 °C. The supernatant was diluted three times with HB buffer. To enrich the membrane fraction, lysates were centrifuged for 90 min at 150,000 × *g*. Finally, the pellet was resuspended in 30 μL 30 mM HEPES and analyzed by western blot.

For coimmunoprecipitation, 1 mg of protein was brought up to 500 μl with lysis buffer (NaCl 150 mM, HEPES 50 mM, Titron X-100 1%, pH 7.4) supplemented with protease inhibitors. Precleaning was performed with 40 μl of protein A Sepharose beads (GE Health care) for 1 h at 4 °C. Next, samples were incubated in a chromatography column (BioRad Micro spin Chromatography Columns), which contained 2.5 μg of anti-GFP antibody (Genescript) previously crosslinked to protein A Sepharose beads, for 2 h at room temperature (RT), with continuous mixing. Next, the columns were centrifuged for 30 s at 1000 × *g*. The supernatant (SN) was kept and stored at − 20 °C. Columns were washed four times with 500 μl of lysis buffer and centrifuged for 30 s at 1000 × *g*. Finally, elution was performed by incubating the columns with 100 μl of 0.2 M glycine pH 2.5 and spun for 30 s at 1000 × *g*. Eluted proteins (IPs) were prepared for western blotting by adding 20 μl of loading buffer (5×) and 5 μl of 1 M Tris–HCl pH 10.

Irreversible crosslinking of the antibody to the sepharose beads was performed by mixing the antibody with protein A sepharose beads for 1 h at RT. Next, the beads were incubated with 500 μl of dimethyl pimelimidate (DMP, Pierce) for 30 min at RT. Columns were washed four times with 500 μl of 1 × TBS, four times with 500 μl of 0.2 M glycine pH 2.5 and three more times with 1 × TBS. Finally, columns were incubated with protein lysates to perform immunoprecipitation, following the protocol described before.

Protein samples (50 μg), SN and IP were boiled in Laemmli SDS loading buffer and separated by 10% SDS–PAGE. Next, samples were transferred to nitrocellulose membranes (Immobilon-P, Millipore) and blocked in 0.2% Tween-20-PBS supplemented with 5% dry milk before immunoreaction. Filters were immunoblotted with antibodies against anti-GFP (1:1,000, Roche), Kvβ1.1 (1/1,000, NeuroMab), Kvβ2.1 (1/1,000, NeuroMab), Kv1.3 (1/200, Neuromab), β-actin (1/50,000, Sigma), ubiquitin (1/500, Santa Cruz), flotillin (1/1,000, BD Transduction), clathrin (1/1,000, BD Transduction), caveolin (1/1,000, BD transduction) or myc (1/1,000, Sigma). Finally, the filters were washed with 0.05% Tween-20-PBS and incubated with horseradish peroxidase-conjugated secondary antibodies (BioRad).

### Confocal microscopy and image analysis

For confocal analysis, cells were seeded on polylysine-coated coverslips 24 h prior to transfection. The next day, the cells were washed twice with PBS without K^+^ (PBS-K^+^), fixed with 4% paraformaldehyde for 10 min, and washed three times for 5 min with PBS-K^+^. Finally, coverslips were mounted on microscope slides (Acefesa) with house Mowiol mounting media. Coverslips were dried at RT for at least 1 day before imaging.

Staining with FITC-labeled cholera toxin β subunit (CTXβ) for lipid raft microdomains and wheat germ agglutinin (WGA)-Texas red (Invitrogen) for the plasma membrane was performed under non-permeabilized conditions [66]. Live cells (on ice) were quickly washed with PBS at 4 °C and stained with a dilution of WGA-Texas Red (1/1,500) in DMEM supplemented with 30 mM HEPES for 10 min at 4 °C. Subsequently, the cells were quickly washed twice and fixed with 4% paraformaldehyde in PBS for 10 min. Next, the cells were washed and mounted as described before. The EEA1 marker was used to stain early endosomes. Fixed cells were further permeabilized with 0.1% Triton X-100 for 10 min. After 60 min of incubation with blocking solution (10% goat serum, 5% nonfat powdered milk and 0.05% Triton X-100), primary mouse anti-EEA1 (1:500) antibody was added at 4 °C overnight (BD Transduction Laboratories) in 10% goat serum and 0.05% Triton X-100. Next, the cells were incubated with secondary goat anti-mouse antibody conjugated with Alexa Fluor 660 for 2 h at RT. Mounting as previously described [27].

All images were acquired with a Leica TCS SL laser scanning confocal spectral microscope (Leica Microsystems) equipped with argon and helium–neon lasers. All experiments were performed with a 63 × oil-immersion objective lens NA 1.32. Colocalization offline image analysis was performed using ImageJ software (https://imagej.nih.gov/ij/). A pixel-by-pixel colocalization study using JACoP (Just Another Colocalization Plugin) was used, and Mander’s overlap coefficient (MOC) was calculated.

### Immunological synapse formation

To analyze the Kvβ2 distribution during IS formation, human B cell lymphoma Raji cells were used as antigen-presenting cells. B cells were incubated with 10 µg/mL *Staphylococcus* enterotoxin E (SEE, Toxin Technologies) for 30 min. Next, Raji cells were mixed with Jurkat T cells (1:1) to form cell conjugates and spun at 200 × *g* for 1 min at 37 °C. Samples were plated on poly-L-lysine-coated coverslips and incubated for 15 min at 37 °C. The cells were washed once with PBS and fixed with 2% PFA in PBS for 10 min. Cells were rinsed three times with PBS between steps. Next, conjugates were labeled with anti-Kvβ2.1 (1/100, NeuroMab), anti-CD3 Alexa 647-conjugated antibody and anti-CD19 Alexa 488-conjugated antibody (1/100, BioLegend). Antibodies were diluted in PBS supplemented with 1% BSA and incubated for 2 h. Coverslips were rinsed and mounted in Mowiol.

Kvβ2 localization at the immune synapse was analyzed using ImageJ software. Briefly, region of interest (ROI) including the whole IS and equal surface areas in both Jurkat and Raji cell membranes outside the IS were drawn. The Kvβ2 signal intensity was measured in each membrane section and the intensity ratio calculated as follows:

$$ ({\text{Kv}}\beta {2}\,\,{\text{intensity at the IS}})/(({\text{Kv}}\beta {2}\,\,{\text{intensity in Jurkat ROI}})\, + \,({\text{Kv}}\beta {2}\,\,{\text{intensity in Raji ROI}})). $$

Therefore, ratios above 1 indicated that Kvβ2 intensities at the IS were higher than the sum of both Kvβ2 intensities in Jurkat and Raji membranes outside IS.

### Cell unroofing preparations (CUP)

HEK-293 cells were seeded in poly-d-lysine-treated glass coverslips. Twenty-four hours after transfection, samples were cooled on ice for 5 min and washed twice in PBS-K^+^. Next, samples were incubated for 5 min in KHMgE buffer (70 mM KCl, 30 mM HEPES, 5 mM MgCl_2_, 3 mM EGTA, pH 7.5) diluted three times and gently washed with nondiluted KHMgE to induce hypotonic shock. Bursted cells were removed from the coverslip by intensive pipetting up and down. After two washes with KHMgE buffer, only membrane sheets remained attached. Preparations were fixed and mounted as previously described [67].

### Lipid-raft isolation

Low-density Triton-insoluble complexes were isolated as previously described [68] from Jurkat T cells and HEK293 cells transiently transfected with Kvβ1.1CFP, Kvβ2.1CFP and the Kvβ2.1CFP^ClessA^ mutant. Cells were homogenized in 1 ml of MBS (150 mM NaCl, 25 mM 2-morpholinoethanesulfonic acid 1-hydrate (MES), pH 6.5) 0.1% Triton X-100, and sucrose was added to a final concentration of 40%. A 5–30% linear sucrose gradient was layered on top and further centrifuged (39,000 rpm) for 20–22 h at 4 °C in a Beckman SW41-Ti rotor. Gradient fractions (1 ml) were sequentially collected from the top of the tube and analyzed by western blotting. While clathrin identified the nonbuoyant fractions, caveolin and flotillin labeled the floating fractions (lipid rafts) in HEK 293 cells and Jurkat T lymphocytes, respectively.

### Ubiquitination assay

Cells were washed twice in cold PBS and frozen at − 80 °C for at least one night. Cells were lysed on ice for 20 min with 2 mL of lysis buffer (50 mM HEPES, 150 mM NaCl, 1% Triton X-100, 10% glycerol, pH 7.5) supplemented with 12.5 mg NEM (Nethylmaleimide, Sigma), 0.2 mM MG132, 1 mM EGTA, 1 mM EDTA, 20 mM NaF, 1% NaOV and 2 mM DTT containing 1 μg/ml aprotinin, 1 μg/ml leupeptin, 1 μg/ml pepstatin and 1 mM phenylmethylsulfonyl fluoride as protease inhibitors. Next, the cells were scrapped and centrifuged at 14,000 × *g* for 15 min. Immunoprecipitation assays were performed as described above.

### Half-life studies

Cells were preincubated with 100 µg/ml cycloheximide (CHX) for 3 h at 37 °C to halt protein synthesis. Next, cells were incubated in the presence or absence of 1 µM PMA at the indicated times. To inhibit lysosomal and proteasomal degradation, 60 nM bafilomycin A1 (BA) or 5 µM MG132 (MG), respectively, was added. Finally, the cells were washed twice with cold PBS, and protein extraction was performed as described above.

### Acyl-biotin exchange (ABE) palmitoylation assay

Detection of protein palmitoylation by acyl-biotin exchange (ABE) was described previously [69]. Briefly, human CD4 + lymphocytes, Jurkat T cells and HEK 293 cells transfected with Kvβ2.1 wild type (WT) and Kvβ2.1 cysteine mutants were washed with cold PBS and harvested in buffer A (150 mM NaCl, 50 mM Tris HCl, 0.2% Triton X-100, 5 mM EDTA, pH 7.4) containing protease inhibitors and 10 mM NEM. Lysates were collected by scraping samples on ice, passed ten times through a 25-gauge needle and incubated for 1 h at 4 °C before centrifugation at 16,000 × *g* for 15 min. Supernatants were chloroform–methanol precipitated, and the pellet was allowed to air dry for 2–3 min. The pellet was resuspended in 300 µl of buffer B (4% SDS, 50 mM Tris HCl, 5 mM EDTA, pH 7.4) supplemented with 10 mM NEM and diluted fourfold in buffer A containing 1 mM NEM. Samples were incubated at 4 °C overnight with gentle agitation. NEM was removed by performing three sequential chloroform–methanol precipitations. Next, the pellet was resuspended in 500 µL of buffer B. The sample was split in two volumes, and 250 µL was diluted with 950 μL of buffer C (50 mM Tris–HCl, 1 mM HPDP-biotin, 0.2% Triton X-100, 100 µM PMSF) containing 0.7 M hydroxylamine (+ HA). The other 250 µL was diluted in buffer C without hydroxylamine (−HA). Samples were incubated at RT with gentle rocking for 1 h. Three rounds of chloroform–methanol precipitation were performed, and the final pellets were resuspended in 300 µL buffer B and diluted with 900 µL buffer D (150 mM NaCl, 50 mM Tris–HCl, 5 mM EDTA, 0.2 mM HPDP-biotin, 0.2% Triton X-100, pH 7.4). Samples were incubated at RT with gentle agitation for 1 h prior to 3 more sequential chloroform–methanol precipitations. The final pellet was resuspended in 120 µL of buffer E (2% SDS, 50 mM Tris HCl, 0,2% Triton X-100, 5 mM EDTA, pH 7.4) and diluted to 0.1% SDS with buffer A. Biotin-labeled proteins were captured with 50 µL of NeutrAvidin agarose beads, previously washed with 400 µL of buffer A and centrifuged for 1 min at 2000 × *g*. NeutrAvidine beads were pelleted by centrifugation at 2,000 × g for 30 s. After washing the beads four times with 400 µL of buffer A, captured proteins were boiled in 100 µL 1 × Laemmli buffer and 2% β-mercaptoethanol. Samples were then analyzed by SDS–PAGE western blot.

### Proximity-ligation-assay (PLA)

Palmitic acid (15-yne) 15-hexadecynoic acid (Avanti Polar Lipids) was used for Alk-C16 protein palmitoylation. Jurkat T lymphocytes and HEK-293 cells expressing Kvβ2.1CFP for 4 h were incubated for 18 h with 100 μM 15-yne and further sonicated for 15 min. Next, the cells were quickly washed three times with PBS-K^+^ and fixed with 2% paraformaldehyde for 10 min, followed by cold methanol for 5 min. After fixation, the cells were washed twice, and a freshly prepared click reaction (0.1 mM biotin-azide (carboxamide-6-azidohexanyl biotin, Invitrogen), 0.1 mM TCEP (Tris(2-carboxyethyl)phosphine hydrochloride, Sigma–Aldrich) and 0.1 mM CuSO_4_ (Sigma–Aldrich)) was added with gentle rocking for 1 h at RT. After five washes with PBS-K^+^, cells were incubated with blocking solution (5% BSA, 0.3% Triton) for 1 h at RT. Cells were further washed three times with PBS-K^+^, incubated with anti-Kvβ2.1 (1:50, Neuromab) and anti-Biotin (1:300, Sigma–Aldrich) antibodies and diluted in Duolink blocking solution 1X (Sigma–Aldrich) at 4 °C overnight. Next, the cells were washed three times and incubated for 1 h at 37 °C with 100 μL of freshly prepared secondary PLA antibodies (20 μL Duolink in situ PLA probe anti-goat MINUS, 20 μL Duolink in situ PLA probe anti-mouse PLUS and 60 μL Duolink antibodies diluent 1X; Sigma–Aldrich). Cells were washed five times and incubated for 30 min at 37 °C with 100 μL ligation-PLA-solution (2.5 U of Duolink ligase, Sigma–Aldrich). After ligation and five further PBS-K^+^ washes, cells were incubated for 100 min at 37 °C with 100 μl amplification-PLA-solution (12.5 U Duolink Polymerase in 1 × Duolink Far Red amplification buffer, Sigma–Aldrich) in the dark. Finally, after four more washes (PBS-K^+^), samples were mounted with in-house Mowiol and dried for 1 day before imaging.

### Supplementary Information

Below is the link to the electronic supplementary material.

Supplementary file1 (PDF 2149 KB)

## Data Availability

The raw data and datasets generated during and/or analyzed during the current study are available from the corresponding author (afelipe@ub.edu) on reasonable request.

## References

[1] Pongs O, Schwarz JR (2010). Ancillary subunits associated with voltage-dependent K+ channels. Physiol Rev.

[2] Capera J, Serrano-Novillo C, Navarro-Perez M, Cassinelli S, Felipe A (2019). The potassium channel odyssey: mechanisms of traffic and membrane arrangement. Int J Mol Sci.

[3] Gulbis JM, Mann S, MacKinnon R (1999). Structure of a voltage-dependent K+ channel beta subunit. Cell.

[4] Rettig J, Heinemann SH, Wunder F, Lorra C, Parcej DN, Dolly JO, Pongs O (1994). Inactivation properties of voltage-gated K+ channels altered by presence of beta-subunit. Nature.

[5] Nagaya N, Papazian DM (1997). Potassium channel alpha and beta subunits assemble in the endoplasmic reticulum. J Biol Chem.

[6] Shi G, Nakahira K, Hammond S, Rhodes KJ, Schechter LE, Trimmer JS (1996). Beta subunits promote K+ channel surface expression through effects early in biosynthesis. Neuron.

[7] Nakahira K, Matos MF, Trimmer JS (1998). Differential interaction of voltage-gated K+ channel beta-subunits with cytoskeleton is mediated by unique amino terminal domains. J Mol Neurosci.

[8] Levin G, Chikvashvili D, Singer-Lahat D, Peretz T, Thornhill WB, Lotan I (1996). Phosphorylation of a K+ channel alpha subunit modulates the inactivation conferred by a beta subunit Involvement of cytoskeleton. J Biol Chem.

[9] Jing J, Peretz T, Singer-Lahat D, Chikvashvili D, Thornhill WB, Lotan I (1997). Inactivation of a voltage-dependent K+ channel by beta subunit. Modulation by a phosphorylation-dependent interaction between the distal C terminus of alpha subunit and cytoskeleton. J Biol Chem.

[10] Gu Y, Gu C (2010). Dynamics of Kv1 channel transport in axons. PLoS One.

[11] Gu C, Zhou W, Puthenveedu MA, Xu M, Jan YN, Jan LY (2006). The microtubule plus-end tracking protein EB1 is required for Kv1 voltage-gated K+ channel axonal targeting. Neuron.

[12] Accili EA, Kiehn J, Yang Q, Wang Z, Brown AM, Wible BA (1997). Separable Kvbeta subunit domains alter expression and gating of potassium channels. J Biol Chem.

[13] Tipparaju SM, Barski OA, Srivastava S, Bhatnagar A (2008). Catalytic mechanism and substrate specificity of the beta-subunit of the voltage-gated potassium channel. Biochemistry.

[14] Xie Z, Barski OA, Cai J, Bhatnagar A, Tipparaju SM (2011). Catalytic reduction of carbonyl groups in oxidized PAPC by Kvbeta2 (AKR6). Chem Biol Interact.

[15] Heilstedt HA, Burgess DL, Anderson AE, Chedrawi A, Tharp B, Lee O, Kashork CD, Starkey DE, Wu YQ, Noebels JL (2001). Loss of the potassium channel beta-subunit gene, KCNAB2, is associated with epilepsy in patients with 1p36 deletion syndrome. Epilepsia.

[16] McCormack K, Connor JX, Zhou L, Ho LL, Ganetzky B, Chiu SY, Messing A (2002). Genetic analysis of the mammalian K+ channel beta subunit Kvbeta 2 (Kcnab2). J Biol Chem.

[17] Connor JX, McCormack K, Pletsch A, Gaeta S, Ganetzky B, Chiu SY, Messing A (2005). Genetic modifiers of the Kv beta2-null phenotype in mice. Genes Brain Behav.

[18] Vicente R, Escalada A, Soler C, Grande M, Celada A, Tamkun MM, Solsona C, Felipe A (2005). Pattern of Kv beta subunit expression in macrophages depends upon proliferation and the mode of activation. J Immunol.

[19] McCormack T, McCormack K, Nadal MS, Vieira E, Ozaita A, Rudy B (1999). The effects of Shaker beta-subunits on the human lymphocyte K+ channel Kv1.3. J Biol Chem.

[20] Panyi G, Varga Z, Gaspar R (2004). Ion channels and lymphocyte activation. Immunol Lett.

[21] Gong J, Xu J, Bezanilla M, van Huizen R, Derin R, Li M (1999). Differential stimulation of PKC phosphorylation of potassium channels by ZIP1 and ZIP2. Science.

[22] Beeton C, Wulff H, Standifer NE, Azam P, Mullen KM, Pennington MW, Kolski-Andreaco A, Wei E, Grino A, Counts DR (2006). Kv13 channels are a therapeutic target for T cell-mediated autoimmune diseases. Proc Natl Acad Sci USA.

[23] Martinez-Marmol R, Styrczewska K, Perez-Verdaguer M, Vallejo-Gracia A, Comes N, Sorkin A, Felipe A (2017). Ubiquitination mediates Kv1.3 endocytosis as a mechanism for protein kinase C-dependent modulation. Sci Rep.

[24] Bi K, Tanaka Y, Coudronniere N, Sugie K, Hong S, van Stipdonk MJ, Altman A (2001). Antigen-induced translocation of PKC-theta to membrane rafts is required for T cell activation. Nat Immunol.

[25] Chandy KG, Norton RS (2017). Peptide blockers of Kv1.3 channels in T cells as therapeutics for autoimmune disease. Curr Opin Chem Biol.

[26] Bahamonde MI, Valverde MA (2003). Voltage-dependent anion channel localises to the plasma membrane and peripheral but not perinuclear mitochondria. Pflugers Arch.

[27] Capera J, Perez-Verdaguer M, Peruzzo R, Navarro-Perez M, Martinez-Pinna J, Alberola-Die A, Morales A, Leanza L, Szabo I, Felipe A (2021). A novel mitochondrial Kv13-caveolin axis controls cell survival and apoptosis. Elife.

[28] Couet J, Li S, Okamoto T, Ikezu T, Lisanti MP (1997). Identification of peptide and protein ligands for the caveolin-scaffolding domain. Implications for the interaction of caveolin with caveolae-associated proteins. J Biol Chem.

[29] Daniotti JL, Pedro MP, Valdez Taubas J (2017). The role of S-acylation in protein trafficking. Traffic.

[30] Webb Y, Hermida-Matsumoto L, Resh MD (2000). Inhibition of protein palmitoylation, raft localization, and T cell signaling by 2-bromopalmitate and polyunsaturated fatty acids. J Biol Chem.

[31] Levental I, Lingwood D, Grzybek M, Coskun U, Simons K (2010). Palmitoylation regulates raft affinity for the majority of integral raft proteins. Proc Natl Acad Sci USA.

[32] Panyi G, Vamosi G, Bacso Z, Bagdany M, Bodnar A, Varga Z, Gaspar R, Matyus L, Damjanovich S (2004). Kv13 potassium channels are localized in the immunological synapse formed between cytotoxic and target cells. Proc Natl Acad Sci USA.

[33] Sanders SS, Martin DD, Butland SL, Lavallee-Adam M, Calzolari D, Kay C, Yates JR, Hayden MR (2015). Curation of the mammalian palmitoylome indicates a pivotal role for palmitoylation in diseases and disorders of the nervous system and cancers. PLoS Comput Biol.

[34] Wan J, Savas JN, Roth AF, Sanders SS, Singaraja RR, Hayden MR, Yates JR, Davis NG (2013). Tracking brain palmitoylation change: predominance of glial change in a mouse model of Huntington’s disease. Chem Biol.

[35] Morrison E, Kuropka B, Kliche S, Brugger B, Krause E, Freund C (2015). Quantitative analysis of the human T cell palmitome. Sci Rep.

[36] Morrison E, Wegner T, Zucchetti AE, Alvaro-Benito M, Zheng A, Kliche S, Krause E, Brugger B, Hivroz C, Freund C (2020). Dynamic palmitoylation events following T-cell receptor signaling. Commun Biol.

[37] Ivaldi C, Martin BR, Kieffer-Jaquinod S, Chapel A, Levade T, Garin J, Journet A (2012). Proteomic analysis of S-acylated proteins in human B cells reveals palmitoylation of the immune regulators CD20 and CD23. PLoS One.

[38] Martin BR, Cravatt BF (2009). Large-scale profiling of protein palmitoylation in mammalian cells. Nat Methods.

[39] Wilson JP, Raghavan AS, Yang YY, Charron G, Hang HC (2011). Proteomic analysis of fatty-acylated proteins in mammalian cells with chemical reporters reveals S-acylation of histone H3 variants. Mol Cell Proteomics.

[40] Davda D, El Azzouny MA, Tom CT, Hernandez JL, Majmudar JD, Kennedy RT, Martin BR (2013). Profiling targets of the irreversible palmitoylation inhibitor 2-bromopalmitate. ACS Chem Biol.

[41] Dart C (2010). Lipid microdomains and the regulation of ion channel function. J Physiol.

[42] Wang X, Zhang J, Berkowski SM, Knowleg H, Chandramouly AB, Downens M, Prystowsky MB (2004). Protein kinase C-mediated phosphorylation of Kv beta 2 in adult rat brain. Neurochem Res.

[43] Shaw AS, Filbert EL (2009). Scaffold proteins and immune-cell signalling. Nat Rev Immunol.

[44] Ludford-Menting MJ, Oliaro J, Sacirbegovic F, Cheah ET, Pedersen N, Thomas SJ, Pasam A, Iazzolino R, Dow LE, Waterhouse NJ (2005). A network of PDZ-containing proteins regulates T cell polarity and morphology during migration and immunological synapse formation. Immunity.

[45] Cho KO, Hunt CA, Kennedy MB (1992). The rat brain postsynaptic density fraction contains a homolog of the Drosophila discs-large tumor suppressor protein. Neuron.

[46] Wong W, Schlichter LC (2004). Differential recruitment of Kv1.4 and Kv4.2 to lipid rafts by PSD-95. J Biol Chem.

[47] O’Neill AK, Gallegos LL, Justilien V, Garcia EL, Leitges M, Fields AP, Hall RA, Newton AC (2011). Protein kinase Calpha promotes cell migration through a PDZ-dependent interaction with its novel substrate discs large homolog 1 (DLG1). J Biol Chem.

[48] Duncan PJ, Bi D, McClafferty H, Chen L, Tian L, Shipston MJ (2019). S-Acylation controls functional coupling of BK channel pore-forming alpha-subunits and beta1-subunits. J Biol Chem.

[49] Charollais J, Van Der Goot FG (2009). Palmitoylation of membrane proteins (review). Mol Membr Biol.

[50] Greaves J, Prescott GR, Gorleku OA, Chamberlain LH (2010). Regulation of SNAP-25 trafficking and function by palmitoylation. Biochem Soc Trans.

[51] Holland SM, Collura KM, Ketschek A, Noma K, Ferguson TA, Jin Y, Gallo G, Thomas GM (2016). Palmitoylation controls DLK localization, interactions and activity to ensure effective axonal injury signaling. Proc Natl Acad Sci USA.

[52] Yang EK, Alvira MR, Levitan ES, Takimoto K (2001). Kvbeta subunits increase expression of Kv4.3 channels by interacting with their C termini. J Biol Chem.

[53] Li Y, Um SY, McDonald TV (2006). Voltage-gated potassium channels: regulation by accessory subunits. Neuroscientist.

[54] Heinemann SH, Rettig J, Graack HR, Pongs O (1996). Functional characterization of Kv channel beta-subunits from rat brain. J Physiol.

[55] Kilfoil PJ, Tipparaju SM, Barski OA, Bhatnagar A (2013). Regulation of ion channels by pyridine nucleotides. Circ Res.

[56] Campomanes CR, Carroll KI, Manganas LN, Hershberger ME, Gong B, Antonucci DE, Rhodes KJ, Trimmer JS (2002). Kv beta subunit oxidoreductase activity and Kv1 potassium channel trafficking. J Biol Chem.

[57] Weng J, Cao Y, Moss N, Zhou M (2006). Modulation of voltage-dependent Shaker family potassium channels by an aldo-keto reductase. J Biol Chem.

[58] Tipparaju SM, Liu SQ, Barski OA, Bhatnagar A (2007). NADPH binding to beta-subunit regulates inactivation of voltage-gated K(+) channels. Biochem Biophys Res Commun.

[59] Croci C, Brandstatter JH, Enz R (2003). ZIP3, a new splice variant of the PKC-zeta-interacting protein family, binds to GABAC receptors, PKC-zeta, and Kv beta 2. J Biol Chem.

[60] Bavassano C, Marvaldi L, Langeslag M, Sarg B, Lindner H, Klimaschewski L, Kress M, Ferrer-Montiel A, Knaus HG (2013). Identification of voltage-gated K(+) channel beta 2 (Kvbeta2) subunit as a novel interaction partner of the pain transducer transient receptor potential vanilloid 1 channel (TRPV1). Biochim Biophys Acta.

[61] Kisselbach J, Schweizer PA, Gerstberger R, Becker R, Katus HA, Thomas D (2012). Enhancement of K2P2.1 (TREK1) background currents expressed in Xenopus oocytes by voltage-gated K+ channel beta subunits. Life Sci.

[62] Roig SR, Sole L, Cassinelli S, Colomer-Molera M, Sastre D, Serrano-Novillo C, Serrano-Albarras A, Lillo MP, Tamkun MM, Felipe A (2021). Calmodulin-dependent KCNE4 dimerization controls membrane targeting. Sci Rep.

[63] Nguyen HM, Miyazaki H, Hoshi N, Smith BJ, Nukina N, Goldin AL, Chandy KG (2012). Modulation of voltage-gated K+ channels by the sodium channel beta1 subunit. Proc Natl Acad Sci USA.

[64] Kubota T, Correa AM, Bezanilla F (2017). Mechanism of functional interaction between potassium channel Kv1.3 and sodium channel NavBeta1 subunit. Sci Rep.

[65] Coppock EA, Martens JR, Tamkun MM (2001). Molecular basis of hypoxia-induced pulmonary vasoconstriction: role of voltage-gated K+ channels. Am J Physiol Lung Cell Mol Physiol.

[66] Sole L, Roura-Ferrer M, Perez-Verdaguer M, Oliveras A, Calvo M, Fernandez-Fernandez JM, Felipe A (2009). KCNE4 suppresses Kv1.3 currents by modulating trafficking, surface expression and channel gating. J Cell Sci.

[67] Oliveras A, Serrano-Novillo C, Moreno C, de la Cruz A, Valenzuela C, Soeller C, Comes N, Felipe A (2020). The unconventional biogenesis of Kv7.1-KCNE1 complexes. Sci Adv.

[68] Martinez-Marmol R, Villalonga N, Sole L, Vicente R, Tamkun MM, Soler C, Felipe A (2008). Multiple Kv1.5 targeting to membrane surface microdomains. J Cell Physiol.

[69] Wan J, Roth AF, Bailey AO, Davis NG (2007). Palmitoylated proteins: purification and identification. Nat Protoc.

