# Supplementary material for: S-acylation-dependent membrane microdomain localization of the regulatory Kvβ2.1 subunit
Source: Cell Mol Life Sci. 2022 Apr 9;79(5):230. doi: 10.1007/s00018-022-04269-3 (PMC8994742; doi:10.1007/s00018-022-04269-3)
Supplement: Supplementary file 1 — Supplementary file1 (PDF 2149 KB) [file 18_2022_4269_MOESM1_ESM.pdf]

S-acylation-dependent membrane microdomain localization of the regulatory Kvβ2.1 subunit

Sara R. Roig[1,2], Silvia Cassinelli[1], María Navarro-Pérez[1], Mireia Pérez-Verdaguer[1,3], Irene Estadella[1], Jesusa Capera[1,4], Antonio Felipe[1].

[1]Molecular Physiology Laboratory, Departament de Bioquímica i Biomedicina Molecular, Institut de Biomedicina (IBUB), Universitat de Barcelona, Avda. Diagonal 643, 08028 Barcelona, Spain. [2]Imaging Core Facility, Biozentrum, University of Basel, 4056 Basel, Switzerland; [3]Department of Cell Biology, School of Medicine, University of Pittsburgh, 3500 Terrace Street, Pittsburgh, PA 15261, United States. [4]Kennedy Institute of Rheumatology, University of Oxford, Oxford OX3 7FY, UK.

*Corresponding author: Antonio Felipe, e-mail address: afelipe@ub.edu

SUPPLEMENTARY INFORMATION

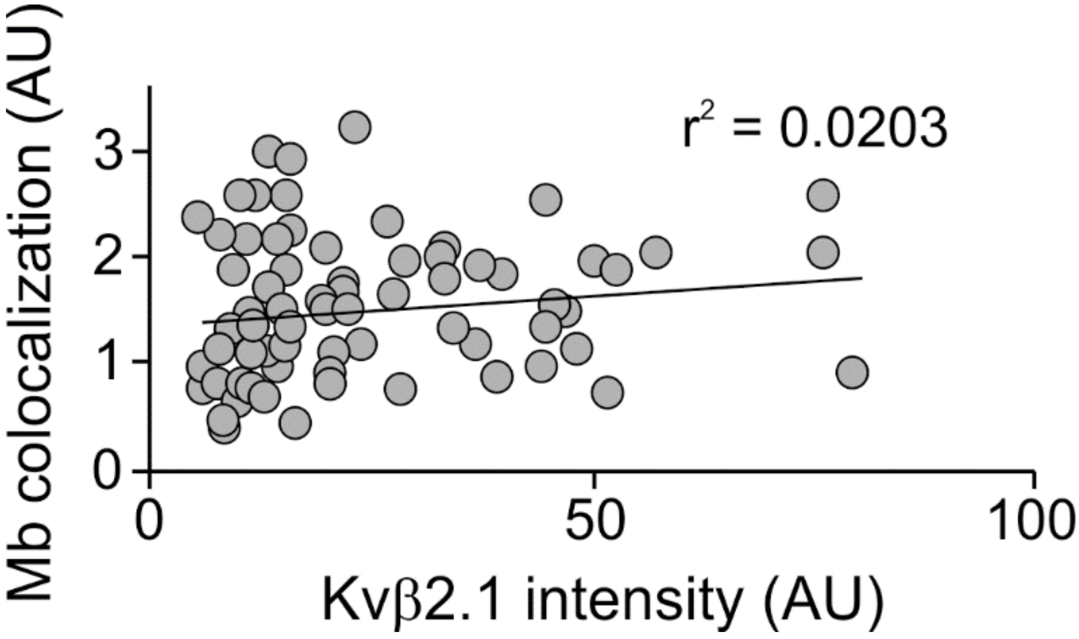

Figure S1. Kvβ2.1 membrane targeting is dose-independent. HEK 293 cells were transfected with Kvβ2.1CFP. The plasma membrane was stained with WGA, and Kvβ2.1 expression and membrane colocalization were measured. Plot of membrane colocalization of Kvβ2.1CFP (Mb) vs. intensity level of Kvβ2.1CFP in the entire cell. A calculated r^2 of 0.0203 indicates no correlation.

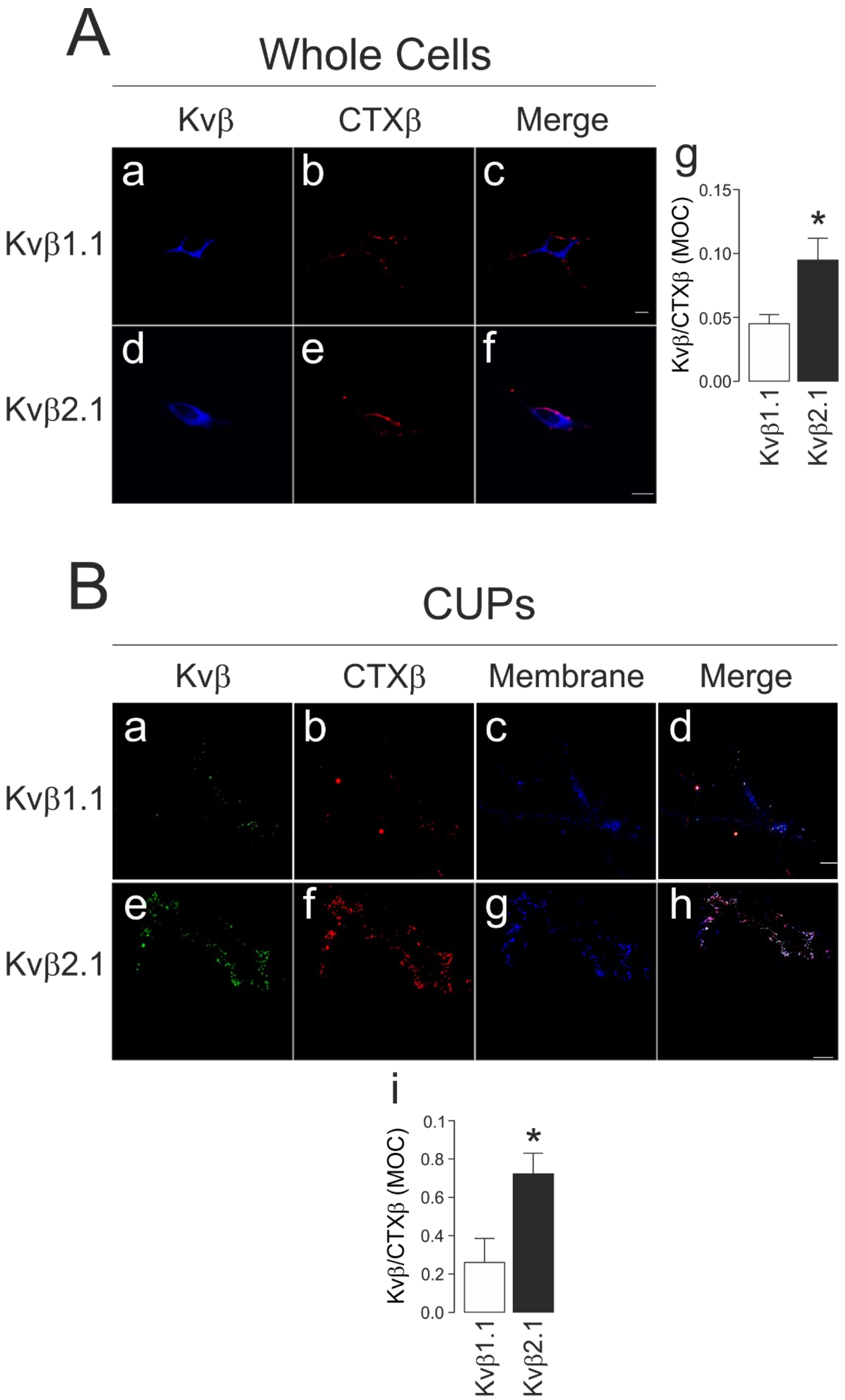
A
Whole Cells
Kvβ
CTXβ
Merge
a
b
c
d
e
f
Kvβ1.1
Kvβ2.1
g
Kvβ/CTXβ (MOC)
0.15
0.10
0.05
0.00
*
Kvβ1.1
Kvβ2.1
B
CUPs
Kvβ
CTXβ
Membrane
Merge
a
b
c
d
e
f
g
h
Kvβ1.1
Kvβ2.1
i
Kvβ/CTXβ (MOC)
0.1
0.8
0.6
0.4
0.2
0.0
*
Kvβ1.1
Kvβ2.1

Figure S2 (previous page). Kvβ2.1 colocalized with CTXβ. Kvβ1.1CFP and Kvβ2.1CFP were expressed in HEK 293 cells. Staining with FITC-labeled cholera toxin β subunit (CTXβ), for lipid raft microdomains, and wheat germ agglutinin (WGA)-Texas red, for the plasma membrane, was performed under non-permeabilized conditions. While whole cell preparations (A) and CUPs (B) were stained with CTXβ, WGA membrane staining was only performed in CUPs. Scale bars represent 10 µm. (Aa-c) Kvβ1.1 in whole cells; (Ad-f) Kvβ2.1 in whole cells. (Ag) Mander's overlap coefficient (MOC) of Kvβ and CTXβ. (Ba-d) Kvβ1.1 in CUPs; (Be-h) Kvβ2.1 in CUPs. (Bi) Mander's overlap coefficient (MOC) of Kvβ and CTXβ. * $p<0.05$ (Student's t-test) vs. Kvβ1.1. Values are mean $\pm$ SE of 20 cells. White bars, Kvβ1.1. Black bars, Kvβ2.1.

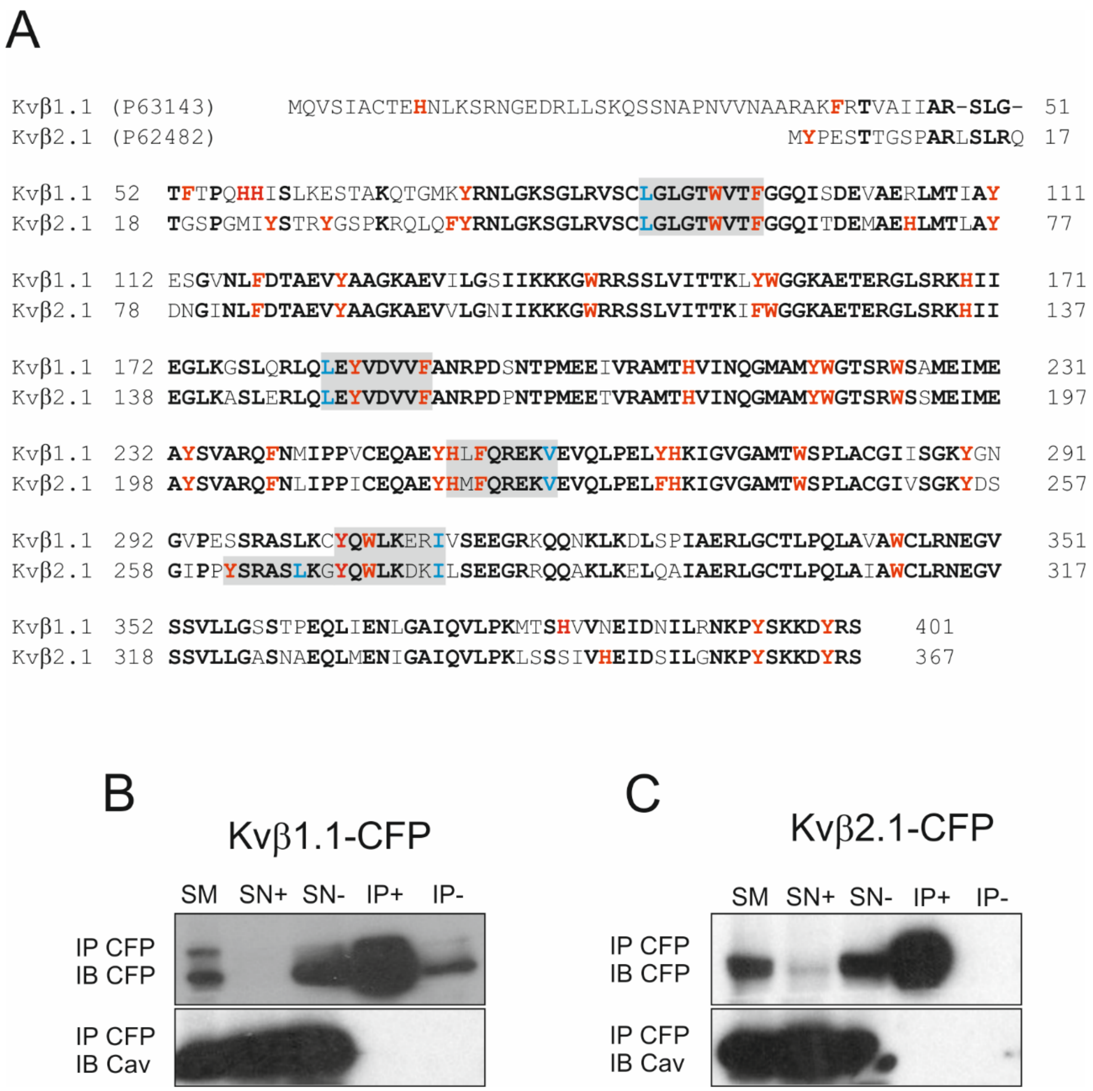

Figure S3. Kvβ2.1 does not interact with caveolin. (A) Amino acid sequence alignment of murine Kvβ1.1 and Kvβ2.1. The UniProt (https://www.uniprot.org/) identification number is indicated in brackets. Sequences were analyzed for putative CBD. Aromatic residues are highlighted in red, and putative hydrophobic CBD clusters are boxed in gray. Alternative hydrophobic residues within clusters are colored in blue. Bold black residues highlight identical amino acids. (B and C) HEK 293 cells were transfected with Kvβ1.1CFP and Kvβ2.1CFP, and the caveolin interaction was analyzed. (B) Kvβ1.1 does not coimmunoprecipitate with caveolin. (C) Kvβ2.1 does not coimmunoprecipitate with caveolin. Lysates were immunoprecipitated (IP) against CFP (Kvβ1.1 and Kvβ2.1) and immunoblotted (IB) against CFP (Kvβ1.1 and Kvβ2.1, top panels) and caveolin (cav, bottom panels). SM, staring materials. SN+, supernatant in presence of antibody. SN-, supernatant in absence of antibody. IP+, immunoprecipitation in the presence of antibody. IP-, immunoprecipitation in the absence of antibody.

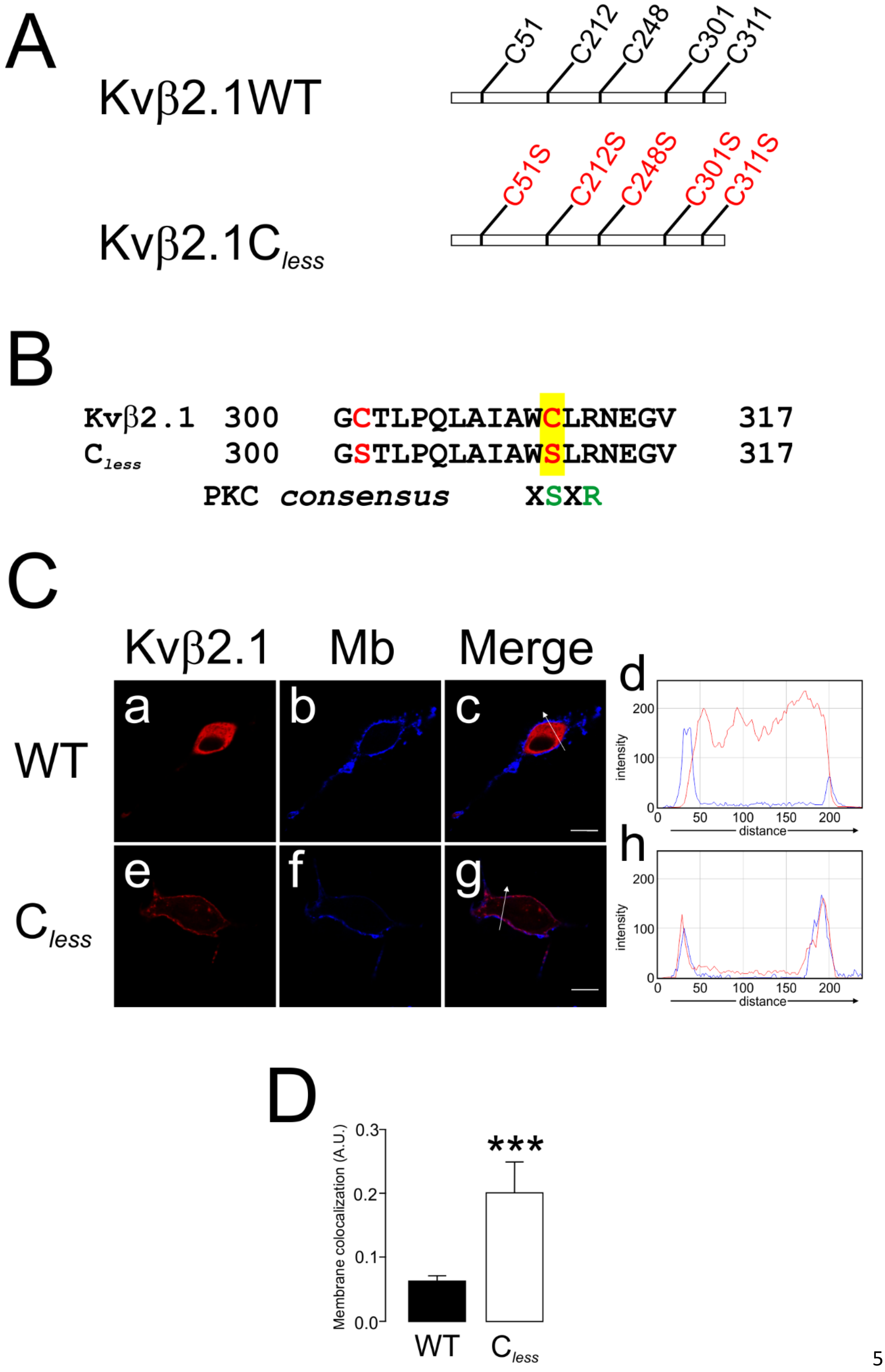
A
Kvβ2.1WT
C51
C212
C248
C301
C311
C51S
C212S
C248S
C301S
C311S
Kvβ2.1C*less*
B
Kvβ2.1 300 GCTLPQLAIAWCLRNEGV 317
C*less* 300 GSTLPQLAIAWSLRNEGV 317
PKC *consensus* XSXR
C
Kvβ2.1
Mb
Merge
WT
C*less*
a
b
c
d
e
f
g
h
intensity
distance
0
50
100
150
200
D
Membrane colocalization (A.U.)
0.0
0.1
0.2
0.3
***
WT
C*less*

Figure S4 (previous page). Schematic representation and aberrant behavior of the mutant Kvβ2.1C_{less}.(A)Representative cartoon. Serine substitutions are shown in red. (B) Sequence analysis of the amino acid residues highlighting the *de novo* introduction of a potential PKC site. (C) Representative images of Kvβ2.1 WT and C_{less} mutant. HEK 293 cells were transfected with Kvβ2.1CFP WT and C_{less} mutant, and the subcellular distribution was analyzed. Kvβ2.1 in red; WGA membrane surface labeling in blue; merge shows colocalization in purple. Panels d and h show the pixel-by-pixel analysis of white arrow sections in c and g, respectively. Scale bars represent 10 µm. (D) Quantification of membrane colocalization using Mander's overlap coefficient (MOC). ***p<0.001 (Student's t-test) vs. WT. Values are mean $\pm$ SE of 30 cells. Black bar, Kvβ2.1 WT; white bar, Kvβ2.1C_{less}.

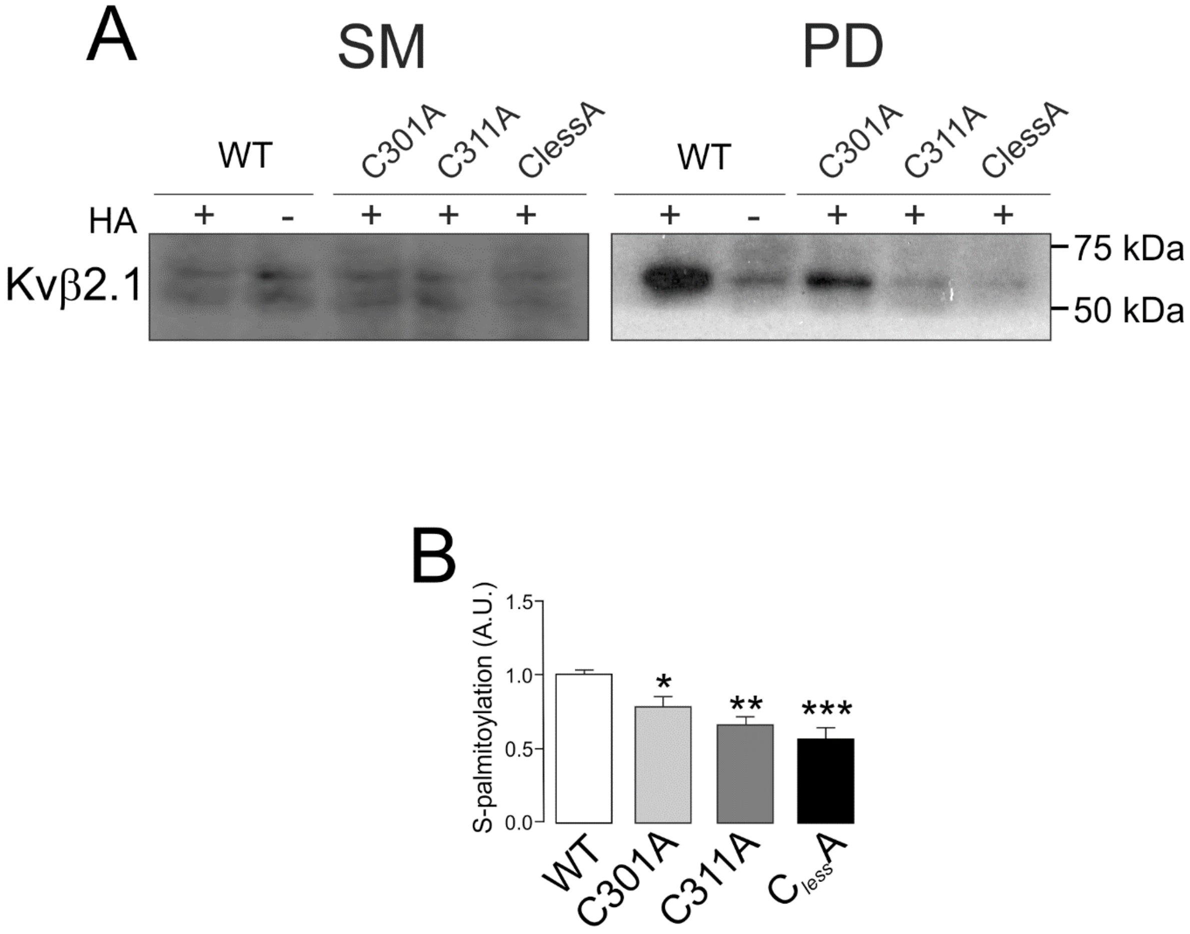

Figure S5. Palmitoylation of Kvβ2.1 WT and single cysteine mutants. HEK 293 cells were transfected with Kvβ2.1CFP WT, single cysteine mutants (C301A, C311A) and C_{less}A. (A) Representative ABE experiment. SM, starting material immunoblotted with an anti-Kvβ2 antibody; PD, palmitoylated pull down. (B) Quantification of pulldowns relative to starting materials. Values are mean ± SE of 3 independent experiments. *, $p<0.05$; **, $p<0.01$; ***, $p<0.001$ (Student's t-test) vs. WT. White bar, Kvβ2.1 WT; light gray bar, C301A; dark gray bar, C311A; black bar, Kvβ2.1C_{less}A.

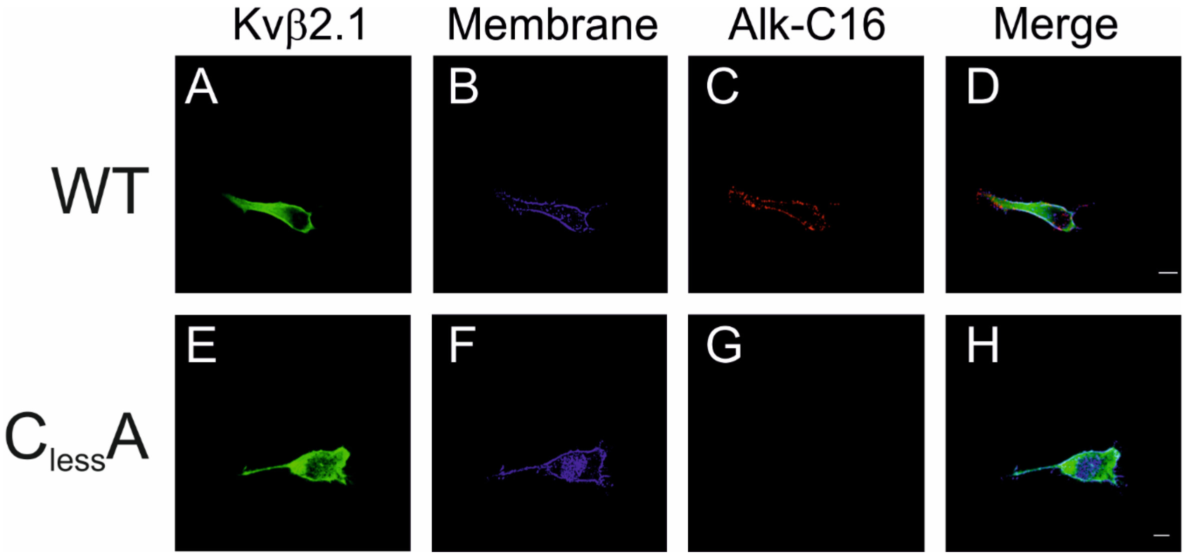

Figure S6. Palmitoylation-dependent cell surface targeting of Kvβ2.1. Proximity-ligation-assay (PLA). Palmitic acid 15-hexadecynoic acid was used for Alk-C16 protein palmitoylation. HEK 293 cells were transfected with Kvβ2.1CFP WT (A-D) and C_{less}A (E-H). (A, E) Total Kvβ2.1CFP in green. (B, F) membrane marker staining in blue. (C, G) Kvβ2.1CFP Alk-C16 palmitoylation in red. (D, H) merge panel highlights Alk-C16 palmitoylation colocalizing with the cell surface. Scale bars represent 10μm.

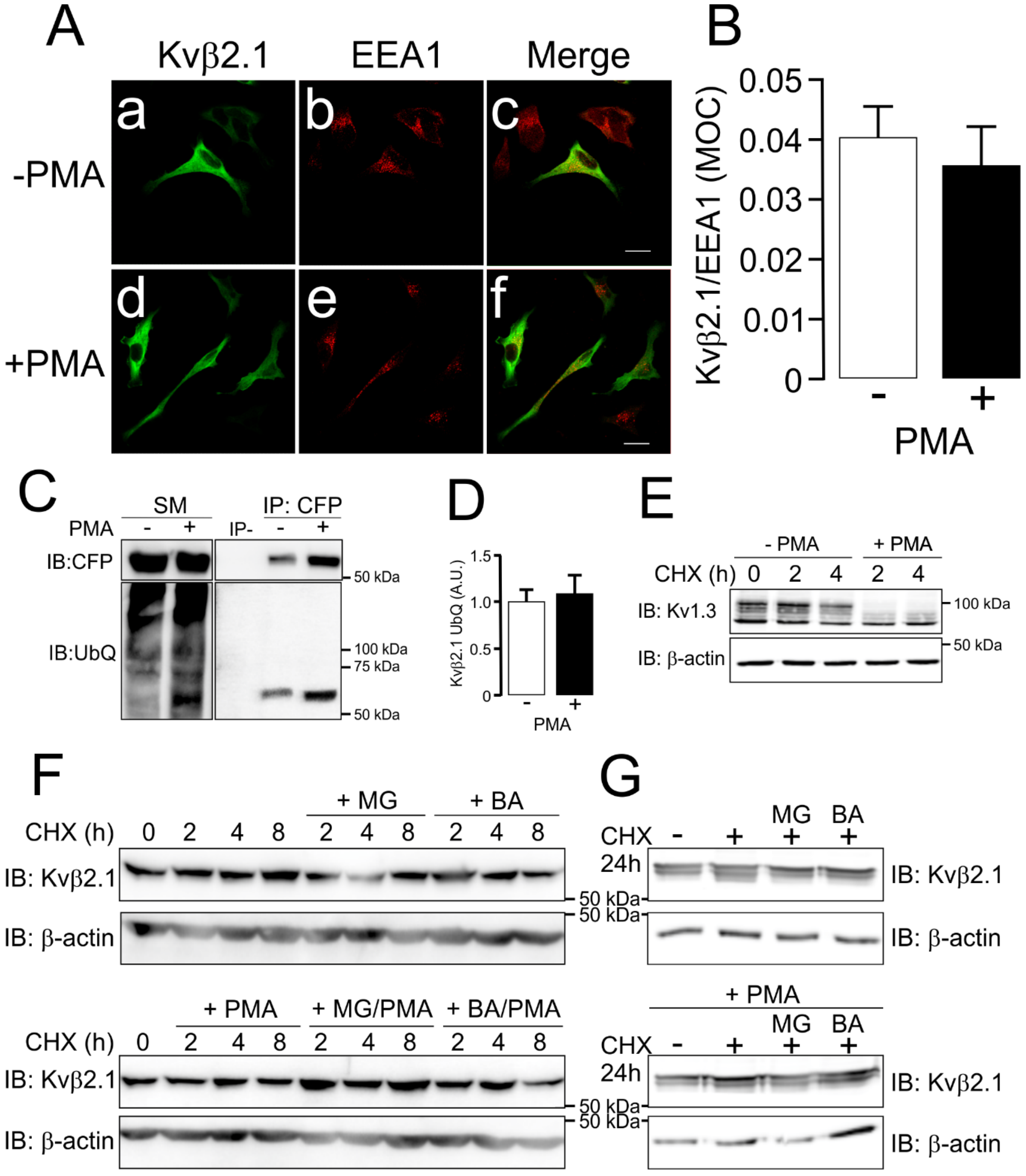

Figure S7. Localization, ubiquitination and stability of Kvβ2.1 upon PMA treatment. HEK 293 cells were transfected with Kvβ2.1CFP, and the early endocytosis location, ubiquitination and degradation fate were analyzed under PMA incubation. (A) Cellular distribution and colocalization with EEA1 of Kvβ2.1 in the absence (-) or presence (+) of PMA. Cells were incubated with PMA for 30 min. Kvβ2.1 CFP in green; EEA1 in red; merged panels, yellow indicates colocalization. Scale bars represents 10 μm. (B)

Quantification of Kvβ2.1/EEA-1 colocalization using Mander's overlap coefficient (MOC). Values are mean $\pm$ SE of 20-30 cells. (C) Kvβ2.1 ubiquitination in the absence (-) or presence (+) of PMA for 30 min. The right panel shows immunoprecipitation (IP) of CFP (Kvβ2.1) and immunoblot (IB) against CFP (top panel) and UbQ (bottom panel). Left panel shows immunoblot (IB) against CFP and UbQ. SM, starting material; IP-, immunoprecipitation in the absence of anti-CFP antibody; IP+, immunoprecipitation in the presence of anti-CFP antibody. (D) Quantification of ubiquitinated Kvβ2.1 upon PMA treatment. Ubiquitinated Kvβ2.1 (IB: UbQ) values, in arbitrary units (A.U.) were relativized to the total immunoprecipitated Kvβ2.1 (IB: CFP). (E) HEK 293 cells were transfected with Kv1.3YFP and its stability was measured in the presence (+) or the absence (-) of PMA at different times (h). Cells were incubated with cycloheximide (CHX) 30 min prior PMA addition and the protein synthesis inhibitor was further present all times. Note that while Kv1.3 abundance slightly decreased at 4 h in the absence of PMA, it almost disappeared as soon as 2 h after PMA treatment. (F) Analysis of the proteasomal and lysosomal degradation of Kvβ2.1 upon PMA treatment at different times (h). Cells were incubated in the absence or presence of MG (MG-132, proteasome inhibitor) and BA (bafilomycin A1, lysosomal inhibitor). (G) Analysis of the Kvβ2.1 stability upon PMA treatment during 24h. Lysates were immunoblotted against Kvβ2.1 (IB: Kvβ2.1). Kvβ2.1 abundancy in the absence (top panels) or presence (bottom panels) of PMA. Note that no Kvβ2.1 changes were observed throughout the experiment.
